# Single neonatal estrogen implant sterilizes female animals by decreasing hypothalamic KISS1 expression

**DOI:** 10.1038/s41598-023-36727-8

**Published:** 2023-06-14

**Authors:** Chan Jin Park, Shiori Minabe, Rex A. Hess, Po-Ching Patrick Lin, Sherry Zhou, Shah Tauseef Bashir, Radwa Barakat, Arnon Gal, CheMyong Jay Ko

**Affiliations:** 1 Department of Comparative Biosciences, College of Veterinary Medicine, University of Illinois at Urbana-Champaign, Urbana, IL 61802 USA; 2 Epivara, Inc, Champaign, IL 61820 USA; 3 Iwate Tohoku Medical Megabank Organization, Iwate Medical University, Iwate, 028-3694 Japan; 4 Department of Toxicology and Forensic Medicine, Faculty of Veterinary Medicine, Benha University, Qalyubia, 13518 Egypt; 5 Department of Veterinary Clinical Medicine, College of Veterinary Medicine, University of Illinois at Urbana-Champaign, Urbana, IL 61802 USA

**Keywords:** Reproductive biology, Infertility

## Abstract

Reproductive sterilization by surgical gonadectomy is strongly advocated to help manage animal populations, especially domesticated pets, and to prevent reproductive behaviors and diseases. This study explored the use of a single-injection method to induce sterility in female animals as an alternative to surgical ovariohysterectomy. The idea was based on our recent finding that repetitive daily injection of estrogen into neonatal rats disrupted hypothalamic expression of Kisspeptin (KISS1), the neuropeptide that triggers and regulates pulsatile secretion of GnRH. Neonatal female rats were dosed with estradiol benzoate (EB) either by daily injections for 11 days or by subcutaneous implantation of an EB-containing silicone capsule designed to release EB over 2–3 weeks. Rats treated by either method did not exhibit estrous cyclicity, were anovulatory, and became infertile. The EB-treated rats had fewer hypothalamic Kisspeptin neurons, but the GnRH-LH axis remained responsive to Kisspeptin stimulation. Because it would be desirable to use a biodegradable carrier that is also easier to handle, an injectable EB carrier was developed from PLGA microspheres to provide pharmacokinetics comparable to the EB-containing silicone capsule. A single neonatal injection of EB-microspheres at an equivalent dosage resulted in sterility in the female rat. In neonatal female Beagle dogs, implantation of an EB-containing silicone capsule also reduced ovarian follicle development and significantly inhibited KISS1 expression in the hypothalamus. None of the treatments produced any concerning health effects, other than infertility. Therefore, further development of this technology for sterilization in domestic female animals, such as dogs and cats is worthy of investigation.

## Introduction

To manage the overpopulation of dogs and cats, as well as other domestic pets, surgical removal of the gonads has long been a routine procedure in veterinary medicine because it produces sterility and eliminates several potential reproductive diseases^1–3^. Reproductive sterilization is preferable to contraception in animals because it is permanent and irreversible; whereas in humans, contraception is preferred because of the desire to control timing of pregnancy, without inducing permanent infertility. Surgical sterilization of animals is well accepted in the United States and other countries, but there has been a long-standing desire for a non-surgical alternative. However, finding an effective, affordable, and humane method has been unsuccessful. The results reported here were obtained by adapting an old chemical, estradiol benzoate (EB), to a new approach of transient neonatal exposure to inhibit kisspeptin neuron development for inducing sterilization in female animals.

Reproduction in mammals is regulated by the hypothalamus-pituitary–gonadal (HPG) axis. An ideal chemical sterilizing agent for animals would be one requiring a single injection that is capable of causing a permanent loss of fertility by targeting a component of the HPG axis. So far, numerous efforts have focused on HPG inhibition using various chemosterilants, hormone analogs and immunocontraception methods to induce sterilization or contraception in domestic pets and food-production animals^4–15^. However, these methods have received limited acceptance, partly because they provide only temporal inhibition, and require repeated dosing to maintain infertility throughout the animal’s reproductive life. In this study, we devised a method that targets neonatal development of the Kisspeptin neurons, with the potential to induce sterility in the female.

Hypothalamic neurons produce two key reproductive hormones, kisspeptin (KISS1) and gonadotropin releasing hormone (GnRH)^16–18^. KISS1, secreted by Kisspeptin neurons, binds to the KISS1 receptor (GPR54) in cell membranes of GnRH neurons, triggering the release of GnRH. GnRH is transported into the pituitary via the portal vein where it stimulates the synthesis and secretion of gonadotropins, luteinizing hormone (LH) and follicle stimulating hormone (FSH), which enter the circulation for delivery to the gonads. LH and FSH stimulate gonadal steroidogenesis, primarily estradiol (E2) production in females, and ultimately regulate growth and release of the germ cells (oocytes)^19^. This stepwise pathway of hormonal stimulation is essential for not only development of the female reproductive system but also for regulatory maintenance of oocyte maturation, ovulation, successful fertilization and pregnancy. Therefore, disruption of the hypothalamic Kisspeptin neuron and/or hypothalamus-pituitary pathway has the potential to impair fertility and even induce sterility. Indeed, gene ablation of *Kiss1,* its receptor *Gpr54,* or the pituitary hormone, LHβ, resulted in hypogonadism and sterility^20–22^, which supports the keen interest in targeting these components of the HPG in the treatment of infertility.

The hypothalamus undergoes sexual differentiation during the neonatal period^23,24^ and exogenous estrogen has been shown to disrupt this process by decreasing the density of Kisspeptin neurons and consequently GnRH neuron activation^25^. A reduction in *Kiss1* expression in the brain of female rats after neonatal treatment with EB was previously reported by Navarro et al.^26^. Recently, we and others^27,28^ found that repetitive daily treatments with EB over a 4–11-day neonatal period substantially inhibited KISS1 protein and mRNA expression in the hypothalamic arcuate nucleus (ARC) region in the female rat. However, repeated injections of a treatment would not be optimal for promoting an alternative to ovariohysterectomy (OVX) in female animals. Therefore, we tested the hypothesis that giving a single neonatal dose of EB in a sustained but temporary release carrier would inhibit Kisspeptin neurons sufficient to induce permanent infertility (sterility), without inducing adverse health effects. Initially, we employed conventional silicone capsules to deliver the EB for a duration exceeding 10 days in rats, and assessed the reproductive capabilities. Then, we formulated poly lactic-co-glycolic acid (PLGA) microspheres to achieve a similar release pattern but in a biodegradable composition. Finally, a long-term goal is to use this type of method for inducing sterility in canines; therefore, a first-step study was performed to determine if neonatal EB would also inhibit KISS1 expression in the female dog.

## Materials and methods

**Figure 1:**
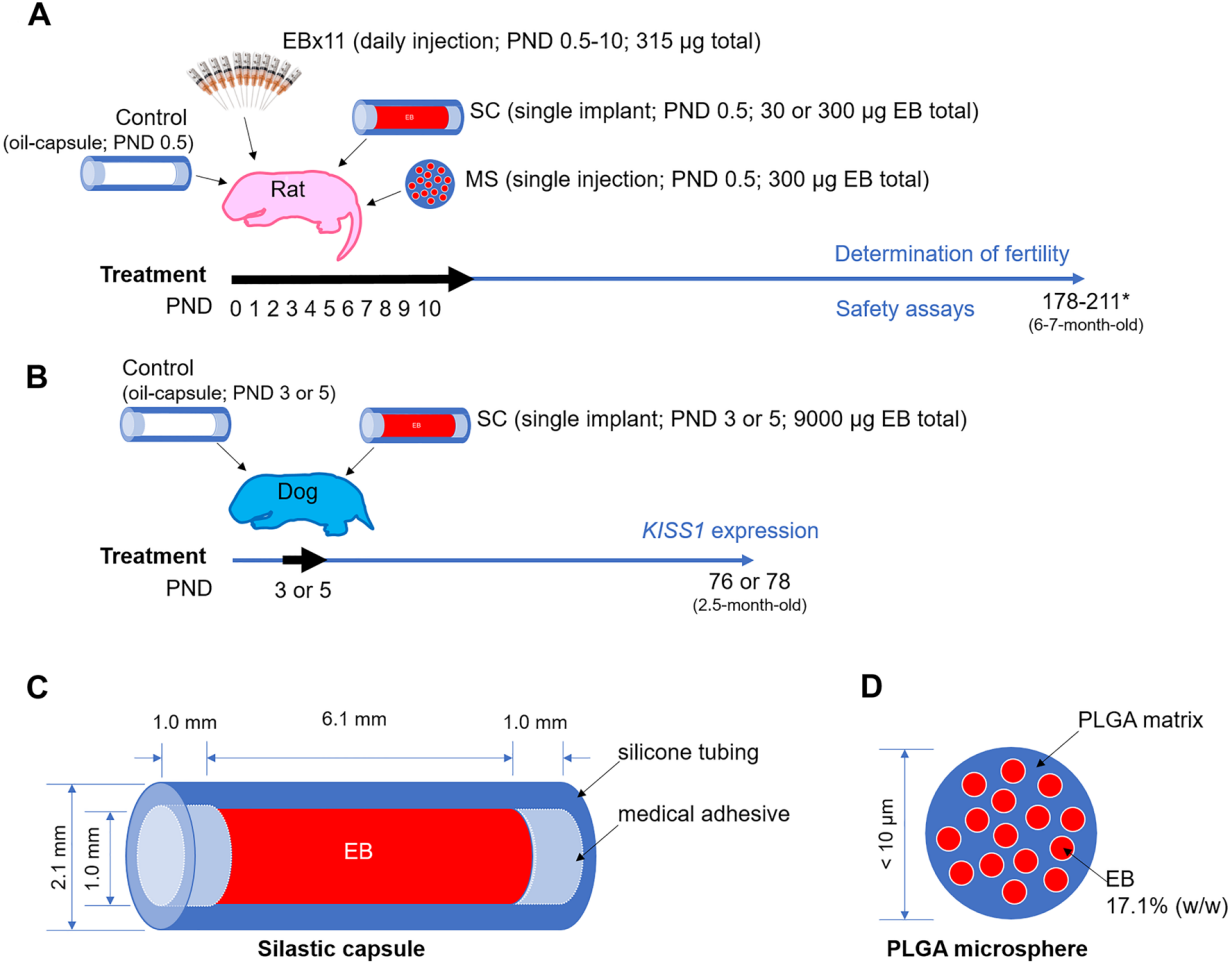
Experimental design. (**A**) Female rats on PND 0.5 were placed into one of the following treatment groups and followed for seven months: control (silicone capsule filled with oil); EBx11 (subcutaneous injection of estradiol benzoate, EB, for 11 consecutive days for a total of 315 µg, PND 0.5–10); SC30 or SC300 (single subcutaneous impant of silicone capsules containing either 30 or 300 µg EB); EBMS300 (single injection of microspheres containing 300 µg of EB. *, some animals kept until PND 335 to determine late vaginal opening (Supplementary Fig. 2). (**B**) Female Beagle dogs received SC9000 (silicone capsules containing 9000 µg EB) on PND 3 or 5, and their hypothalamic *KISS1* expression was compared to that of control groups’ at PND 78. (**C**) Implants were made of silicone capsules with an inner diameter of 1.0 mm filled with EB and endcaps of silicone adhesive. (**D**) Biodegradable microspheres (EBMS300) consisted of matrix of poly lactic-co-glycolic acid (PLGA) forming spheres having less than 10 µm diameter and physically entrapping EB at 17.1% concentration.

Neonatal female rats were used for the initial studies, with all treatments given on the day of birth (postnatal day [PND] 0.5). In the first study, controls (silicone capsule with oil) were compared to a single injection of EB (in silicone capsule for slow release) and to daily injections of EB (in oil) over 11 days (Fig. 1). However, we found the silicone capsules complicated to prepare and handle and because they were permanently embedded under the skin, EB-loaded PLGA microspheres were developed as an alternative biodegradable solution that could be given by a single injection.

### Animals

Sprague Dawley rats used in this study were purchased from Charles River Laboratory and bred at the University of Illinois Division of Animal Resources and maintained under controlled lighting (12 h light/12 h dark) with continuous access to food and water. Rats were acclimatized for one week prior to beginning the experimental procedures. Breeder pairs were housed together for 14 days, with pregnant females then being housed individually, and pups were weaned at 21 days of age (Fig. 1). The rats were euthanized by CO_2_ asphyxiation followed by cervical dislocation, as approved by the University of Illinois Animal Care and Use Committee.

Neonatal female Beagles (*canis lupus familiaris*) were purchased and housed at a Class R research facility, Ridglan Farm (Ridglan Inc., Blue Mounds, WI). Dogs maintained under controlled lighting (12 h light/12 h dark). Newborn puppies received their mother’s milk until weaning. The pups were fed a commercially available feed and water was available ad libitum from automatic waterers. Dog were monitored daily during the study period to determine undesired side effects of treatment. They were euthanized at PND 76–78 by intravenous injection of Fatal Plus^®^ (0.04 mg/kg; Vortech Pharmaceuticals, Dearborn, MI) (Fig. 1), as approved by the Ridglan Animal Care and Use Committee.

### EB-capsules and EB-microspheres

Silicone capsules (SC) were fabricated using Silastic™ tubing (Cat. 508-005, Dow Corning, Midland, MI) pre-cut to length (volume calculated from the inner diameter) to hold sufficient dosage, with an extra 1 mm on each end for sealing with medical silicone adhesive^29,30^. EB (Cat. #E8515, Sigma, St. Louis, MO) was mixed with sesame oil (Cat. S3547, Sigma) and loaded by syringe into the silicone capsules at a total dosage of either 30 or 300 μg and sealed with the adhesive.

EB-loaded PLGA microspheres (EBMS) were synthesized by SpineThera (Minneapolis, MN), with the final product of EBMS being in a dry powder form (Lot 6028–119, SpineThera; 17.1% EB, D_50_ = 3.64 µm, D_90_ = 5.48 µm, D_99_ = 8.72 µm) that was then reconstituted with PBS solution (pH 7.4) to 50 μl for injection by syringe.

### Rat experimental groups

Several female rat experiments were performed using the following different treatment groups (A–D):

A. Control (Silicone capsule with sesame oil vehicle only, implanted on PND 0.5);
B. EBx11(Daily subcutaneous injections, given for 11 consecutive days for an EB total approximately 48 mg/kg BW, in 50 µL of sesame oil, using a dose of 10 µg on PND 0.5 to 3, 25 µg on PND 4 to 6, and 50 µg on PND 7–10)^27^;
C. SC300 (High-dose single injection of EB in silicone capsule containing 300 μg EB in sesame oil, implanted on PND 0.5 at approximately 46 mg/kg BW);
D. SC30 (Low-dose single injection of EB in silicone capsule containing 30 μg EB in sesame oil, implanted on PND 0.5 at approximately 4.6 mg/kg BW).

The injections and implants were placed subcutaneously in the back of the nape area^31^. Impantation was performed by skin incision with a sterile 10-gauge needle or surgical scissors after wiping the skin with Betadine^®^ disinfectant solution. The incision was sealed with tissue adhesive (Fig. 1). For some endpoints only control and SC300 groups were used (E2 concentrations; gene expressions) or control, EBx11 and SC300 groups (Body and organ weights; mammary gland morphology; liver histopathology). In all experiments, the female neonatal (newborn) rats were divided randomly into the respective treatment groups using Research Randomizer (https://www.randomizer.org/). The number of animals used per treatment group is listed for each experiment and associated endpoints. For some endpoints, there are unequal numbers between groups, which was due to tissue sample availability, animal terminations for short-term studies and the number of female rats born for inclusion in a specific trial.

### Rat experiment 1: fertility study; EB treatment by single capsule versus multiple injections

Fertility of the female rats was determined at PND 180–210 (6–7 months), a time point that would represent the middle age of their reproductive life span. Female controls (n = 11), EBx11 (n = 4), SC300 (n = 8), and SC30 (n = 4) were housed continuously with proven breeder males for 14 days and fertility outcomes determined 21 days later. Percent fertility was calculated as the number of females that gave birth divided by the total number of females per group in each round of tests. Some rats, control (n = 6) and SC300 (n = 5) were kept until PND 335 (11-month-old) to determine if a late vaginal opening had occurred.

### Rat experiment 2: short-term study, ovarian histology and serum hormones

Experiment 2 included four treatment groups: Control (n = 7), EB ×  11 (n = 3), SC300 (n = 4), SC30 (n = 3). On PND 60–70 (2–2.5 months), blood was collected from all animals by tail vein puncture for serum E2 and testosterone measurements (described below) and the females were euthanized on PND 75. The ovaries and uteri were collected for histological evaluation (described below).

### Rat experiments 3 and 4: long-term study, body weight, estrous, vaginal opening, organ weights and histology, femur bone measurement, serum chemistry and hormone analysis

Experiment 3 included four treatment groups: Control (n = 8), EBx11 (n = 6), SC300 (n = 5), SC30 (n = 9). Experiment 4 included two treatment groups: Control (n = 6) and SC300 (n = 5), For some endpoints, data were combined from both experiments: body and organ weights, serum chemistry, CBC and hormone measurements. On PND 178–211 (6–7 months), body weights were determined and the stage of estrous cycle and vaginal opening were evaluated for each animal. Blood was then collected for serum chemistry, CBC and hormone measurements (described below) and the females were then euthanized. The anovaginal distance (AVD) was measured and the major organs removed and weighed. The ovaries, the uteri and livers were collected for histological evaluation and mammary gland tissues were collected for whole mount analysis (described below). Blood cell population (CBC) was analyzed from 1 mL whole blood that was collected by cardiac puncture using BD Vacutainer^®^ Plus blood collection tubes coated with a spray-dried K2 EDTA (Becton Dickinson, Franklin Lakes, NJ, USA). Immediately after collection, samples were gently inverted several times and complete blood count (CBC) analyses were conducted at the University of Illinois Veterinary Medicine Diagnostics Laboratory (Urbana, IL).

### Rat experiment 5: in vivo serum and hypothalamus E2 concentration profiles

Experiment 5 included two treatment groups: Control and SC300. The rats were euthanized at each time point for sample collection. Serum E2 levels were measured on 0, 1, 2, 3, 4, 7, 10, 21, and 30 days after treatment (PND 0.5) to determine the normal physiological E2 levels and the pharmacokinetics of E2 release in the SC300 implant. The concentration of E2 in the hypothalamus of the same rats were measured on 0, 1, 2, 3, 4, 7, and 10 days after treatment (methods described below). Respective numbers of animals per group are found in Supplementary Table 1.

### Rat experiment 6: immunolocalization of KISS1 in the rat hypothalamus

Experiment 6 included two treatment groups, Control and SC300, and two time points post treatment, PND 29 (n = 3 for each group) and PND 75 (n = 3 for each group). The rats were euthanized and the hypothalamus tissues carefully removed and fixed for immunohistological evaluation of KISS1 expression (methods described below). Control rats were in diestrus stage of cycle on PND 75 based on vaginal cytology. After the animals were euthanized, a bilateral thoracotomy was performed and the vasculature was immediately perfused with ice cold PBS (100–200 ml), followed by 200–300 ml freshly prepared 4% PFA. The fixed brains were carefully removed from the skull without damaging the hypothalamus and post-fixed overnight in 4% PFA at 4 °C. After fixation, the tissues were washed with PBS and transferred to 30% sucrose containing 0.1% sodium azide (at 4 °C for 2–3 days until the brain sinks). The samples were washed with PBS and placed in OCT filled plastic tissue molds and frozen by placing sections in dry ice and ethanol slushy and then stored at − 80 °C. Tissues were cryosectioned at 50 μm and every fourth section was collected. Sections that contained the ARC were identified and stored at 4 °C in PBS + 0.1% sodium azide solution. Before starting the staining process, rabbit anti-KISS1 (a gift from Dr. Hitoshi Ozawa of Department of Anatomy and Neurobiology, Nippon Medical School, Tokyo Japan; RRID:AB_2910199) was pre-adsorbed overnight with Neuropeptide FF (NPFF; 10 ug/ml) in 5% normal serum + 1% BSA dissolved in PBS + 0.1% sodium azide + 0.3% Triton-X (hereafter known as blocking buffer) as described previously^32^. Brain slices were treated with blocking buffer for 1 h and then incubated in 1 ml pre-adsorbed primary antibody solution (1:1000). After 72 h of incubation with primary antibody, sections were washed 3 times with 5–10 ml PBS for 10 min. Next, 1:750 goat anti-rabbit Alexa fluor 488 plus (Invitrogen) in blocking buffer 1–2 ml was added and incubated for 2.5 h followed by addition of DAPI (Invitrogen) (1:100,000) for 30 min. Stained sections were washed 3 times with 5–10 ml PBS for 10 min. A final wash with 5 ml TBS for 10 min removed PBS precipitate. During all steps of blocking, incubating and washing, the sections were rocked gently on a shaker at RT. Brain sections were mounted on glass slides and air-dried before adding ProLong™ Gold Mount media (Invitrogen), coverslipped and cured for 24 h at RT in the dark. Sections were imaged using a confocal microscope (A1, Nikon, Japan). KISS1 immunoreactivity (KISS1-ir) was quantified relative to DAPI signal (nuclear staining) in confocal images from PND 75 rats. All images were subjected to the same threshold to remove any background. In each image, a ratio of total DAPI to Alexa fluor 488-positive pixels was calculated using Image J (NIH) to give a relative expression of KISS1 in each image. An average of 7–8 images were normalized to control data. To remove bias, a blinded pixel analysis was performed.

### Rat experiment 7: in vivo KP-10 challenge

To determine if the animals were responsive to Kisspeptin following treatment, experiment 7 included two treatment groups: Control (n = 4) and SC300 (n = 3). On PND 60–70 (2–2.5 months) after treatment on PND 0.5, peripheral blood (50 µL from the ventral tail vain) was drawn and serum collected and immediately afterwards the females were injected intraperitoneally with a Kisspeptin-10 analog (KP-10; 50 nmole, Millipore-Sigma, San Diego, CA). After 30 min, blood was drawn again and serum collected. Serum LH concentration was measured by a sandwich immunoassay using monoclonal antibodies against both bovine LH (Cat. 581B7, Medix Biochemica, Kauniainen, Finland) and human LH-beta subunit (Cat. 5303, Medix Biochemica), at the University of Virginia Center for Research in Reproduction Ligand Assay and Analysis Core, as previously described (range 0.04–37.4 ng/mL)^33^. Blood cell population in control and treated rats were analyzed by collecting 1 mL whole blood by cardiac puncture using BD Vacutainer^®^ Plus blood collection tubes coated with a spray-dried K2 EDTA (Becton Dickinson, Franklin Lakes, NJ, USA).

### Rat experiment 8: hypothalamus single-cell analysis

*Single-cell RNA-seq library preparation and library preprocessing*. To investigate differential gene expression in individual cells of the rat hypothalamus, experiment 8 included two treatment groups: Control (n = 3) and SC300 (n = 3), with tissue collection on PND 29. After animal euthanasia, hypothalamus tissues were collected and pooled by treatment group. A total of 4,049 cells from Control and 4,223 cells from SC300-treated females were obtained. For single-cell RNA sequencing (scRNAseq) analyses, tissues were dissociated by collagenase as previously described^34^. Briefly, hypothalamic tissues that contain AVPV and ARC area were cut into small pieces, transferred to 2 ml of collagenase digestion solution containing 3.5 U of collagenase type I (17100-017; Invitrogen, Carlsbad, CA), 1000 U of deoxyribonuclease I (D4527; Sigma), and 40 mg of BSA (017K0723; Sigma) in H-199 media (12350-039; Life Technologies, Inc., Carlsbad, CA), then placed in a water bath at 37 C for 30 min. After incubation, tissues were further dissociated by repeatedly passing them through an 18-G needle attached to a 3-ml syringe. Next, digested tissue suspension was filtered through a 40-μm filter, centrifuged at 300 × *g* for 5 min, then resuspended in 1 ml of PBS containing 1% BSA. Single-cell suspensions with a viability of > 90% by Trypan Blue staining on the TC-20 (Bio-Rad Laboratories, Inc. CA) were converted into individually barcoded cDNA libraries with the Single-Cell 3’ Chromium kit version 3 from 10 × Genomics (Pleasanton, CA), following the manufacturer’s protocols. The 10 × Chromium instrument separates thousands of single cells into Gel Bead Emulsions (GEMs) that add a barcode to the mRNA from each individual cell. Following ds-cDNA synthesis, individually barcoded libraries compatible with the Illumina chemistry were constructed. The final libraries were quantitated on Qubit and the average size determined on the AATI Fragment Analyzer (Advanced Analytics, Ames, IA). Libraries were pooled evenly, and the final pool was diluted to 5 nM concentration and further quantitated by qPCR on a Bio-Rad CFX Connect Real-Time System (Bio-Rad Laboratories, Inc. CA). The final library pool was sequenced on a 2 × 150nt S4 lane in a NovaSeq 6000. Basecalling and demultiplexing of raw data was done with the mkfastq command of the software Cell Ranger 3.0.2 (10 × Genomics). Single-cell expression was initially analyzed to perform quality control, sample de-multiplexing, barcode processing, and single-cell 3′ gene counting. Sequencing reads were aligned to the Ensembl96 transcriptome using the Cell Ranger suite with default parameters. Samples were merged using Cellranger aggregate function with default parameters. A total of 4,488 and 4,524 cells from hypothalami of Control and SC300 rats, respectively, was analyzed. Mean raw reads per cell were 151,484 and 147,555 in each sample. Median numbers of expressed genes per cell were 2479 and 2530 in each sample. Further analysis was performed in R (v3.5) using the cellrangerRkit (v2.2.0), Seurat package (v3.0.0), and Monocle package (v2.8.0)^35,36^.

*Single-cell RNA-seq data analysis*. Analysis of single-cell RNA-seq was performed using the Seurat package^35^. Feature-barcode matrices from each data set were imported using Read10 × function and CreateSeuratObject function. For each cell, a minimum expression of 200 genes was applied to filtered uninformative cells. For each gene, a minimum of expressions from 3 cells was applied. After log-normalization using NormalizeData function, highly variable genes were detected on the basis of the average expression and dispersion per gene using the FindVariableFeatures function with default parameters. Dimensionality reduction and visualization for the 10 × data was performed using Uniform Manifold Approximation and Projection (UMAP) for cells^37^. The first 30 principal components of high-variance genes were used as input for the UMAP algorithm with the following default settings. Clustering on the UMAP embedding was performed using FindClusters function with ‘resolution = 0.7’. Cells were labeled by their group name, Control and SC300. Hypothalamic cell types were determined by known cell type-specific marker genes (Supplementary Fig. 1). In the PND 29 hypothalamic tissues of pooled Control and SC300 groups, GABAergic neurons (*Snp25*^+^*Slc32a1*^+^), Glutamatergic neurons (*Snap25*^+^*Slc17a6*^+^), astrocytes (*Agt*^+^), oligodendrocytes (*Pdgfra*^+^, *Fyn*^+^, or *Mobp*^+^), microglia cells (*Tmem119*^+^), ependymal cells (*Tmem212*^+^), non-neuronal cells (*Olig1*^+^), stromal cells (*Mgp*^+^), and macrophages (*Pf4*^+^) were identified. Cell type-specific differential gene expression was determined by FindMarkers function and visualized by Featureplot and Vlnplot. Differential gene expression between clusters was tested by default 'bimod' likelihood ratio test using FindMarkers function (cut-off, avg_logFC > 0.25, avg_logFC < − 0.25, p_val_adj < 0.05)^35,38^.

### Rat experiment 9: treatment of EB-loaded PLGA microspheres (EBMS) in rats

In this experiment, it was hypothesized that biodegradable microspheres with embedded EB could be easier to handle for giving precise dosages to animals, compared to silicone capsules, and that EB delivery in this form would have the same inhibitory effect on female reproduction. Therefore, biodegradable poly(lactic-*co*-glycolic) acid (PLGA) microspheres with embedded EB (EBMS) were developed for single injection. This experiment included two treatment groups: Control (n = 8) and EBMS300 (300 µg EB) (n = 8). Blank PLGA microspheres without EB were unavailable; therefore, untreated females were used as controls. The EBMS powder was reconstituted with PBS solution (pH 7.4) to provide an average EB dosage of 46 mg/kg body weight. Neonatal animals were treated on the same day as in the previous rat experiments (PND 0.5) by subcutaneous injection in the back of the nape area.

To assess the impact on reproductive status, including the onset of puberty and fertility, treated female rats were examined for vaginal opening and estrous cycle stage on PND 45 (1.5 months). Fertility was tested in two rounds of breeding. The females were housed with proven breeder males from PND 54 to 105. Then on PND 120 (4 months) the rats were euthanized and the ovaries were collected for histological evaluation (described below). Ovarian tissue volumes were calculated by measuring the length and width using a caliper.

### Dog experiment

The purpose of this study was to collect preliminary data regarding the potential translation of the rat experiments for the use of neonatal EB treatment in domesticated animal species to replace surgical OVX of the females. The goal of the experiment was to determine if *KISS1* expression was inhibited in the hypothalamus, as in the rat and whether this inhibition would affect growth of the dog ovaries. One litter of neonatal female Beagles was implanted on PND 3 with silicone capsules containing only oil vehicle (Control; n = 2) or silicone capsules containing 9,000 μg EB (SC9000; 22.5 mg/kg BW; n = 2). Two dogs from another litter were treated on PND 5 (Control, n = 1; SC9000, n = 1) and data from both litters were combined (n = 3) for analysis. The implants were placed subcutaneously in the nape of the puppies. Given the observed decrease in *KISS1* expression in rats treated with EB at around one month of age, we euthanized the dogs at PND 76–78 (2.5 months), which corresponds to one month in rats^39^, and collected their ovaries and hypothalami. Ovaries were immediately fixed in 4% paraformaldehyde (PFA) for 24 h and processed for histological observation (methods described below). Hematoxylin and eosin-stained ovary slides were analyzed to compare follicle status between the control group and the SC9000 group. The hypothalamus was dissected after carefully removing the brain from the skull. Total RNA was extracted from the isolated hypothalmic tissues and analyzed for *KISS1* expression using Reat-time PCR (methods described below).

### Estrous cycle, vaginal opening and anovaginal distance in rats

The stage of the estrous cycle was ascertained by conducting vaginal cytology from female control (n = 8), EBx11 (n = 6), SC300 (n = 5), and SC30 (n = 9) between PND 62 and 69. Aqueous vaginal smears were applied to glass microscope slides and inspected using light microscopy to identify the estrous cycle stage. The vaginal opening was observed in same rats from PND 28 to 74. When rats exhibit complete canalization of the vagina, it was determined as complete vaginal opening. Anovaginal distance was measured using a caliper.

### Hormone measurements and complete blood counts in rats

For the measurement of serum hormone levels and E2 concentration in the hypothalamus, rats were euthanized for each sample collection. Peripheral blood was collected at each time-point by cardiac puncture. The blood was centrifuged at 2000 × *g* for 10 min, and serum collected and preserved at − 20 °C until analyzed. Hypothalamic tissues were isolated, weighted, and homogenized in methanol. Steroids were recovered using diethyl ether according to lipid-lipid extraction methods^40–42^. The measurement results and the number of animals for each time point are provided in Supplementary Table 1. E2 Sensitive ELISA kit (Cat. EIA4399, DRG Diagnostic, Mountainside, NJ) with a reportable range of 1.40–200 pg/mL was used to measure circulating 17β-E2. Testosterone ELISA kit (Cat. EIA1559, DRG Diagnostic) with a reportable range of 0.083–16 ng/mL was used to measure circulating Testosterone. The intra- and inter-assay coefficients of variability were less than 10%.

### Histology and histopathology

After euthanasia, the organs for histological evaluation (described in the respective experiments) were dissected and fixed in 4% paraformaldehyde (PFA) for 24 h, washed in 70% ethanol, processed automatically, and embedded in paraffin for sectioning at 7 μm. Tissue slides were stained with hematoxylin and eosin. Liver histopathology was performed only for control and SC300-treated rats to assess any potential liver toxicity or related adverse effects related to EB treatment. Hematoxylin and eosin-stained liver slides were evaluted by a board-certified veterinary pathologist (A.G.) according to the International Harmonization of Toxicologic Pathology Nomenclature guidelines^43^. For evaluation of the mammary gland, abdominal mammary tissues were processed for whole mount study as previously described^44^. The tissue was fixation in Carnoy’s solution, stained with Carmine alum solution, followed by an acidic ethanol wash^44^ and mounted on glass slides. The slides were imaged with an Olympus BX51 microscope (Melville, NY) to determine mammary tissue changes in branching and terminal end bud formation.

### Real-time PCR

To determine hypothalamic *KISS1* expression, hypothalamic tissues isolated from female dogs were subjected to RT-PCR analysis. Total RNA was extracted using RNAqueous^®^-Micro Kit (Ambion^®^ by Life Technologies™, Carlsbad, CA) according to the manufacturer’s protocol. RNA concentrations were measured with a NanoDrop 1000 spectrophotometer (Thermo Scientific, Waltham, MA). The RNA was diluted to equal concentrations before being reverse transcribed using a high-capacity cDNA reverse transcription kit (Applied Biosynthesis, Foster City, CA). PCR reactions were performed with Power SYBR^®^ Green PCR Master Mix (Applied Biosystems) according to the manufacturer’s protocol. Target specific PCR primers, Gapdh-F: 5’-GTGAAGGTCGGAGTGAACG-3’, Gapdh-R: 5’-AGGGGTCATTGATGGCGAC-3’, Kiss1-F: 5’-GGGTGCCACCTTTTCTAATGTC-3’, and Kiss1-R: 5’-GGTTTCTTGGCAGCTAATGCT-3’ were used for quantification of dog *GAPDH* (housekeeping; product size: 100 bp) and *KISS1* (product size: 70 bp) mRNA levels. Fluorescence was measured using the ABI prism 7500 quantitative real-time thermocycler (Applied Biosystems). Results are expressed as fold differences in relative gene expression with respect to control or equivalent group.

### Statistics

Data analyses were performed using Microsoft Excel, GraphPad Prism (v9.3.1), and SPSS (v18.0). Continuous data were analyzed for normal distribution by a Shapiro–Wilk test. All normally distributed continuous data were analyzed with parametric tests (Student’s *t*-test or ANOVA). Data are graphically presented as the mean ± standard deviation (SD) or ± standard error (SE), and statistically significant differences were accepted when *P*-value is lower than 0.05.

### Ethics statement

This study was carried out in accordance with the recommendations in the Guide for the Care and Use of Laboratory Animals of the National Institutes of Health and ARRIVE guideline^45^. The rat study protocols were approved by the University of Illinois Animal Care and Use Committee (Protocols: 20027). The dog study protocol was reviewed and approved by Institutional Animal Care and Use Committee of Ridglan Farms, AAALAC accredited research facility (Protocol: IN-100-001), and all efforts were made to minimize animal suffering.

## Results

### EB-capsule implant elevated blood and hypothalamic E2 levels in neonatal rats

**Figure 2:**
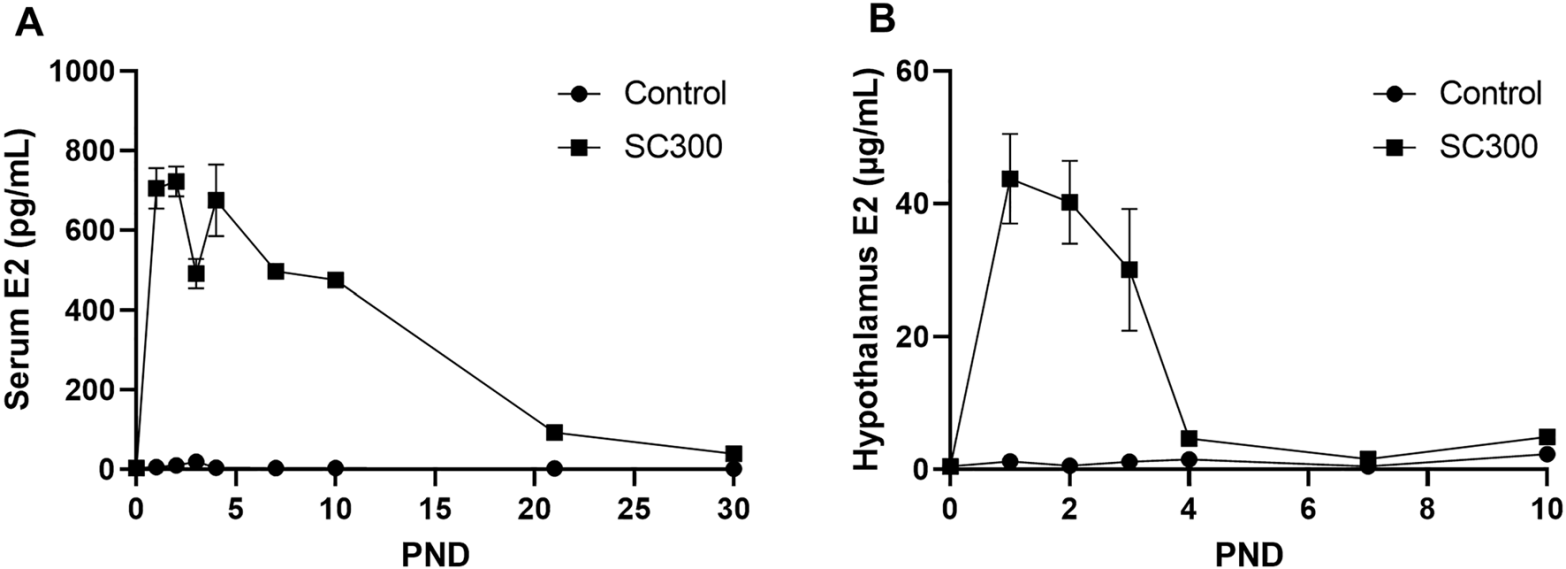
Estradiol (E2) concentrations in serum and hypothalamus after estradiol benzoate (EB) capsule implantation. (**A**) E2 levels in the sera (pg/mL) from PND 0.5 to PND 30. (**B**) E2 levels in homogenized hypothalamic tissue (µg/mL) from PND 0.5 to PND 10. The number of animals for each date are provided in Supplementary Table 1. Error bars = SD. SC300 = 300 μg EB.

A normal low level of serum E2 was observed in control neonatal rats (< 5 pg/ml) from PND 1 to 30 (Fig. 2). In SC300 treated neonatal rats, serum E2 levels were elevated to 712.3 ± 81.3 pg/mL (mean ± SD) by day 1, decreased to 496.7 ± 86.6 pg/mL by day 7, and then further decreased to 33.6 ± 8.5 pg/mL by PND 30, which was higher than in control rats (2.8 ± 2.7 pg/mL). E2 was also measured in isolated hypothalamic tissues in the same rats from PND 1 to 10. Hypothalamic E2 concentrations in SC300 were elevated on day 1 (44.1 ± 9.6 µg/mL) through day 3 (31.3 ± 14.6 µg/mL), but then the level dropped sharply, reaching the control group level by day 7 (1.8 ± 0.4 µg/mL) (Supplementary Table 1). These results indicate that the SC300 implant maintained supraphysiologic levels of E2 for 30 days in circulation, but for only 3–4 days in the hypothalamus.

### EB-capsule implant altered reproductive organ development

**Figure 3:**
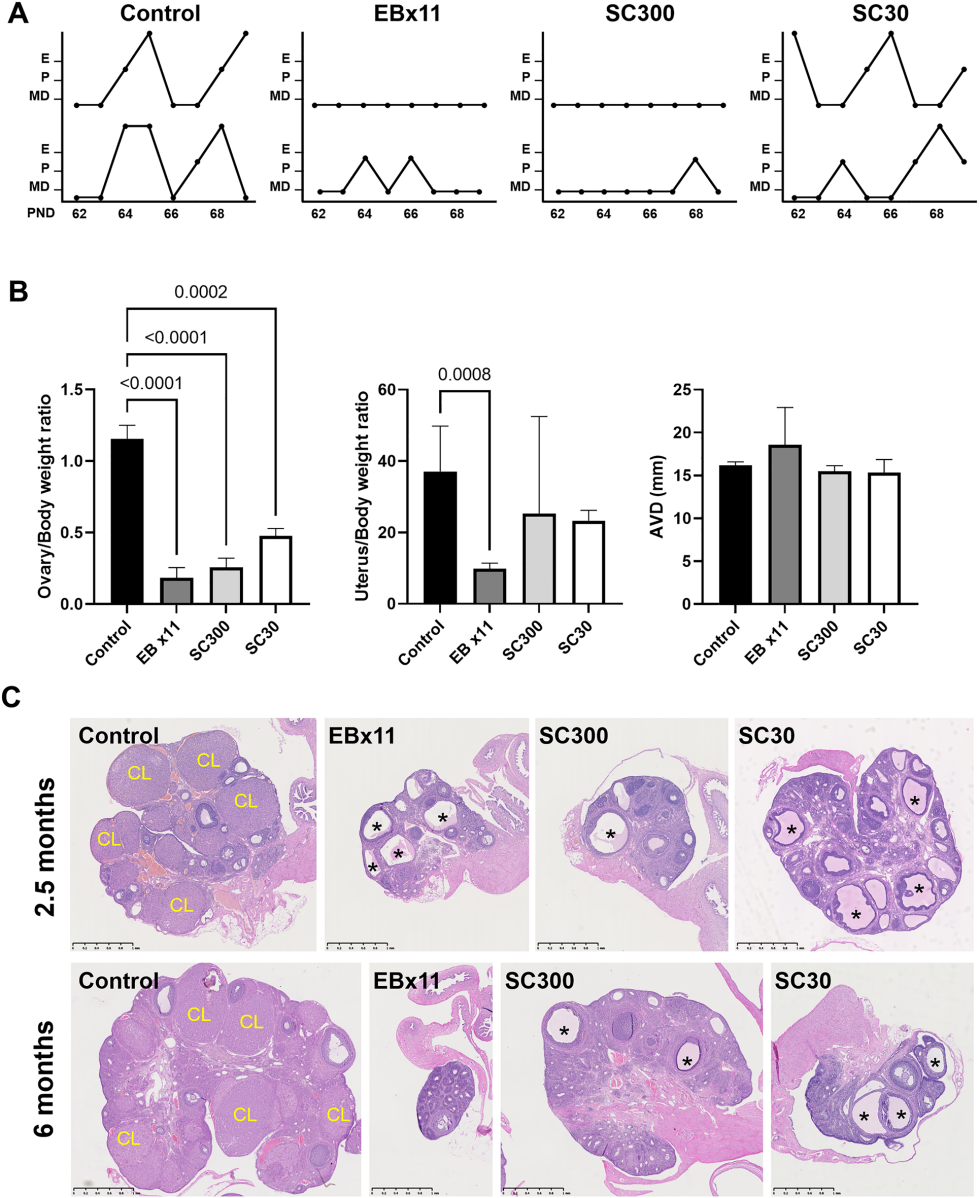
Neonatal estradiol benzoate (EB) inhibited reproductive organ development and ovarian function in female rats. Controls received silicone capsules with oil only. The EBx11 group received EB injections on 11 consecutive days from birth (total of 315 µg EB). The EB300 and EB30 groups received a single implant of a silicone capsule containing 300 or 30 µg EB (SC300 or SC30), respectively. (**A**) Estrous cyclicity in representative rats in each group from PND 62 to PND 69. E, estrus; P, proestrus; MD, met-diestrus. (**B**) Ovary weight/body weight ratio; uterus weight/body weight ratio by EB treatment at PND 178–211 (6–7 months), and Anovaginal distance (AVD; mm) (Control, n = 14; EBx11, n = 6; SC300, n = 10; SC30, n = 9). Error bars = SD. There was a significantly lower ovary/body weight ratio in all EB treatment groups compared to control; *P-*values indicate significant differences (One-way ANOVA with Tukey post hoc test). (**C**) Representative histology of control ovaries at PND 60–75 (2.5 months) (Control, n = 7; EBx11, n = 3; SC300, n = 4; SC30, n = 3) or PND 178–211 (6–7 months) (Control, n = 8; EBx11, n = 6; SC300, n = 5; SC30, n = 9) show numerous corpora lutea (CL). In the EB treated groups, the ovaries have no corpus luteum, but numerous anovulatory follicles (*) are present.

After the rat reached pubertal age (PND 62-69), controls had normal estrous cycles and vaginal openings, while all SC300 exhibited no sign of estrous cycle (Fig. 3A) and had an incomplete vaginal opening (incomplete canalization of the vagina) (Supplementary Fig. 2). The low dose (SC30) rats displayed an anestrus phase and normal vaginal opening during the same observation period.

At 6–7 months (PND178-211), the ovaries were smaller in all treated rats (EBx11, SC300, and SC30) compared to the controls. The EBx11 rats had smaller uteri than the controls, while the SC300 and SC30 rats had uteri similar in size to controls (Fig. 3B). There was no difference in AVD between the groups. The ovaries were examined for histopathological changes at both 2.5 months (PND 75) and 6–7 months (PND178-211). Control ovaries had follicles of various developmental stage and multiple corpora lutea that form after ovulation has taken place. In contrast, while the ovaries of the EBx11, SC300, and SC30 treated rats had all stages of follicles including antral follicles, they did not have corpus luteum formations, indicating an absence of ovulation (Fig. 3C). Thus, neonatal EB treatment did not impair folliculogenesis, but rather inhibited ovulation. No sign of ovulation was seen at mid-reproductive age (6–7 months) in all three EB treatment groups as well, indicating that the neonatal EB treatment permanently impaired the ovulatory machinery. At 11 months of age, the vagina remained incompletely opened in the SC300 females (Supplementary Fig. 1), indicating the lack of maturation in external reproductive organs.

### Neonatal EB-capsule implant induced sterility

The effects of neonatal EB treatment on fertility were compared between controls and implanted EB-capsules (high and low doses) and daily subcutaneous injections over 11 days. The females were housed with proven-fertile males for 14 days and all EB-treated rats were infertile, regardless of dosage or type of EB delivery (Table 1). The control rats exhibited fertility, with 58.3% producing litters with an average of 14 pups/litter; however, in this study the controls had a reduced fertility compared to the historical normal (see Table 2 for comparison). Regardless, none of the females in the EBx11, SC300, and SC30 treatment groups produced offspring.

**Table 1:** Fertility in female rats after neonatal estradiol benzoate (300 μg) in silicone capsule (SC) treatment.

Treatment	Control	EBx11	SC300	SC30
Treatment age	PND 0.5	PND 0.5–10	PND 0.5	PND 0.5
n	11	4	8	4
Age (months)	6-7	6-7	6-7	6-7
Fertility	6/11	0/4	0/8	0/4
Fertility (%)	54.5	0*	0*	0*
Pups/litter	12.2 ± 1.7^a^	0^b^	0^b^	0^b^

Control, capsule containing vehicle only, implanted on PND 0.5; EBx11, EB-treated for 11 consecutive days from birth (total of 315 µg); EB300 (300 μg) or EB30 (30 μg), single EB-capsule-implanted on PND 0.5.

*Significantly different from the control (*P* < 0.001, Fisher’s exact test).

^a^mean ± SD.

^b^Significantly different from the control (*P* < 0.001, One-way ANOVA with Tukey post hoc test).

**Table 2:** Fertility in female rats after neonatal estradiol benzoate (300 μg) in microspheres (EBMS) treatment.

Group	n	First round (PND 24–29)		Second round (PND 54–105)	
		Fertility (%) (Birth/Rat × 100)	Fecundity (Pups/Litter)	Fertility (%) (Birth/Rat × 100)	Fecundity (Pups/Litter)
Control	8	87.5%	12.4 ± 1.0	87.5%	10.8 ± 1.0
EBMS300	8	0%*	0^a^	0%*	0^a^

*Significantly different from control (P < 0.001, Fisher’s exact test).

^a^Significantly different from control (P < 0.001, Student’s t-test).

### Neonatal EB-capsule implant inhibited hypothalamic Kisspeptin neurons

**Figure 4:**
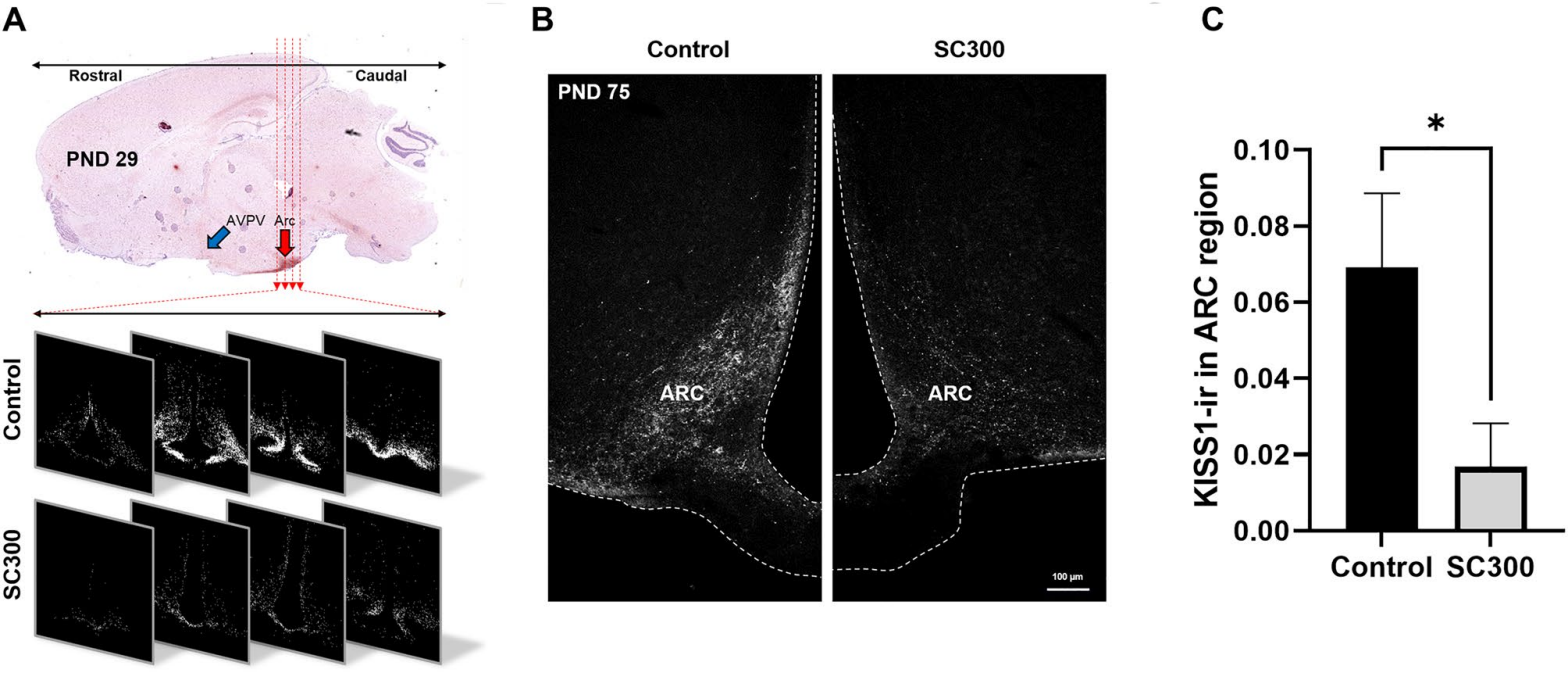
Effects of neonatal estradiol benzoate (EB) on KISS expression in the ARC region of the hypothalamus. (**A**) The top photo displays the AVPV and ARC regions of the hypothalamus in a sagittal section of the brain, illustrating the areas where the KISS1-immunofluorescence images were captured. In the bottom panel, serial sections from the ARC region reveal immunofluorescence for KISS1 in both control and SC300 rats on PND 29, with lower immunoreactivity in the SC300 sections. (**B,C**) KISS1-ir in ARC region of PND75 rats in control and SC300 (300 μg EB) groups. In B, the image of the ARC region in a rat treated with SC300 exhibited lower KISS1-ir than in the control. In C, quantification of the KISS1-ir revealed a significant decrease in the SC300 rats compared to the control group. Error bars = SD (n = 3). *P-value < 0.05 (Student’s *t*-test).

**Figure 5:**
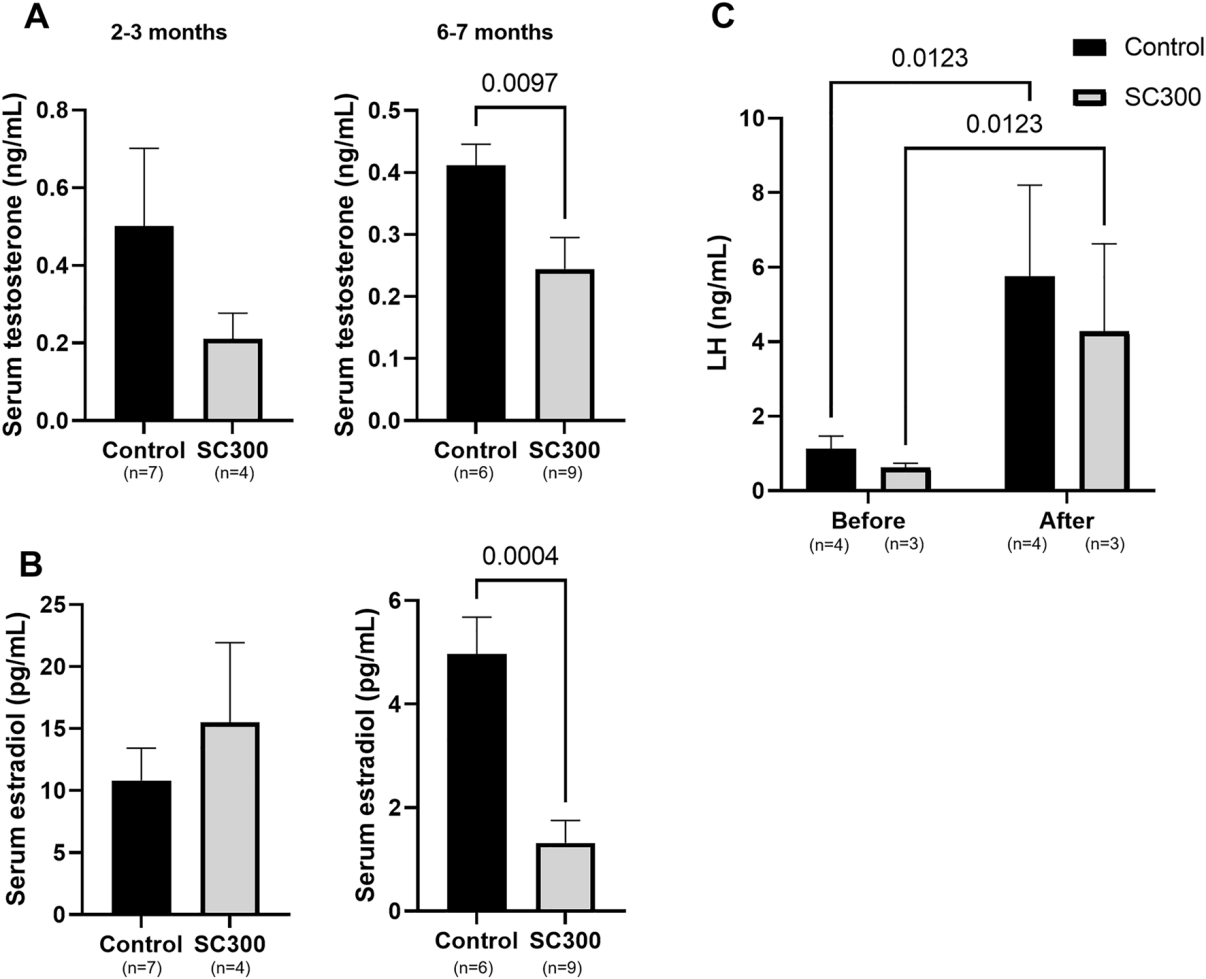
Serum testosterone and estradiol levels in adult control and SC300 (300 μg estradiol benzoate) treated rats and the effect of KP-10 injection on serum LH. Serum concentrations of (**A**) testosterone and (**B**) estradiol in PND 75 (2.5 months of age) rats (Control, n = 7; SC300, n = 4) and PND 178–211 (6–7 months of age) rats (Control, n = 6; SC300, n = 9). Significant differences between the Control and SC300 are indicated by *P*-values (Student’s *t*-test). (**C**)**.** Serum LH levels in control (n = 4) and SC300 (n = 3) rats before and after the treatment of kisspeptin analog, KP-10. After intraperitoneal injection of KP-10 (50 nmole/animal) in rats at 2–2.5 months of age, serum LH levels at 30 min were significantly increased, compared to baseline before injection. Significant differences are indicated by *P*-values (Two-way ANOVA with Tukey post hoc test). Error bars = SD.

A major goal of this study was to develop a single injection method to induce sterility by inhibiting the development of Kisspeptin neurons, which would then indirectly inhibit ovarian function. Therefore, KISS1 expression was assessed in the ARC region of the hypothalamus by intensity of KISS1-ir. Results showed significantly lower KISS1-ir in the ARC of SC300 rats on PND 29 (Fig. 4A) and PND 75 (Fig. 4B and C), compared to controls. Serum testosterone levels (E2 precursor) did not differ from the controls at PND 75 (2.5 months), but were lower at PND 178–211 (6–7 months) (Fig. 5A). Similarly, SC300 serum E2 levels (ovarian activity) were not significantly different at 2.5 months but were lower at 6–7 months compared to control (Fig. 5B). In the KP-10 challenge, KP-10 injection produced a robust increase in serum LH in both control and SC300 groups, indicating that the GnRH neurons of the hypothalamus and pituitary gonadotrophs were still intact and capable of responding to the KISS1 signal (Fig. 5C**).**

### Neonatal EB-capsule implant decreased the number of Kisspeptin neurons

**Figure 6:**
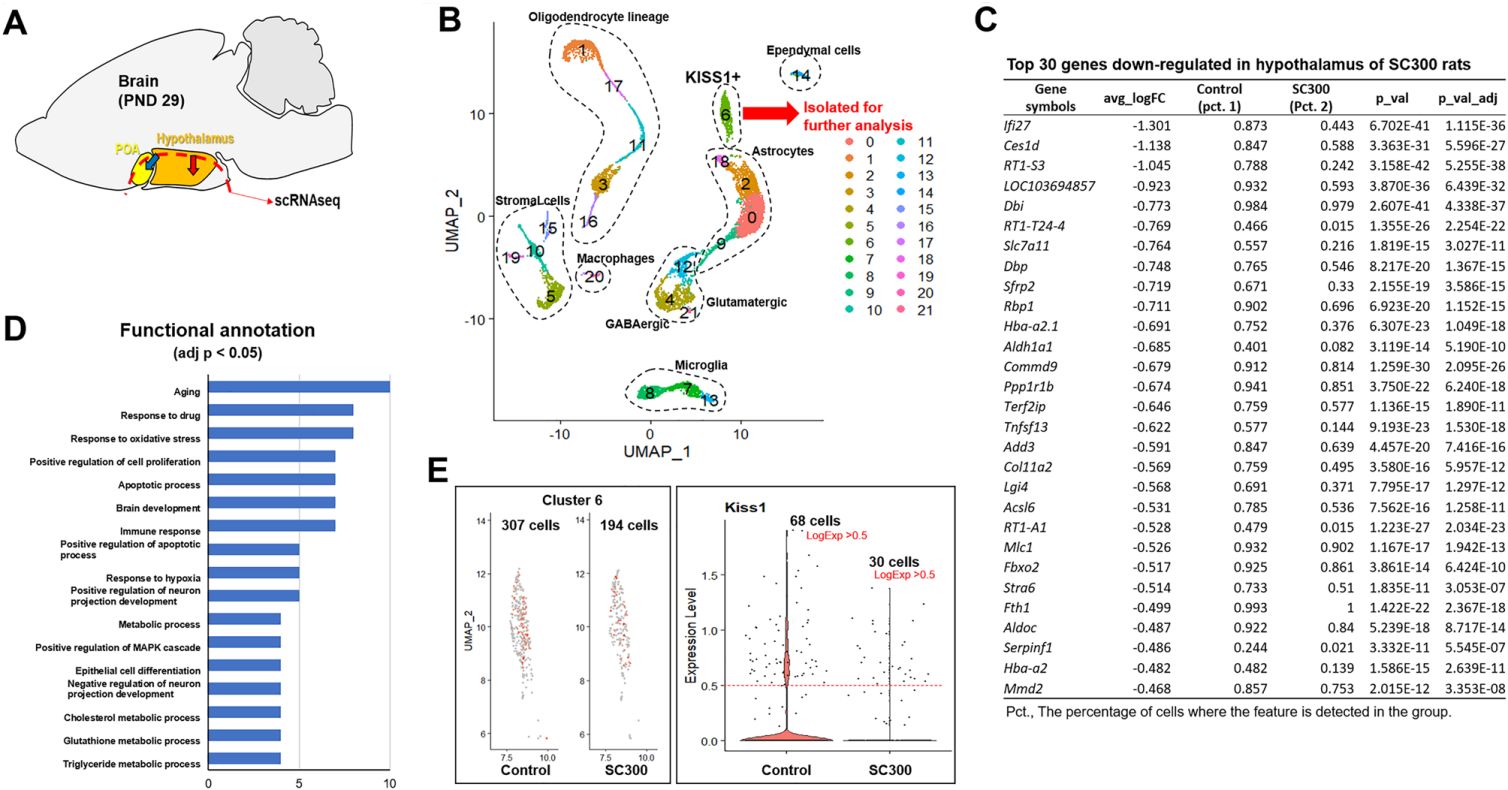
Effects of neonatal estradiol benzoate (EB) on gene expression profiles of *Kiss1*-clusters in Control and SC300 hypothalami. (**A**) The hypothalamic area used for scRNAseq is marked by dotted line. (**B**) Pooled hypothalamic cells of Control (4,049 total) and the SC300 group (4223 total) were UMAP clustered together. Cells were identified by marker gene expressions. Cluster 6 was used for identifying genes that were differentially expressed in SC300 and Control. (**C**) Down-regulated genes in the hypothalamus of SC300 compared to Control are listed with log fold-change of the average expression between the two groups (avg_logFC), percentage of cells where the feature is detected in the first group or second group (Pct. 1 or Pct. 2), P-value (p_val), and adjusted P-value (P_val_adj). (**D**) Down-regulated genes in SC300 are grouped according to functional categories. X-axis indicates the number of DEGs in each category. (**E**) The first panel shows the total number of cells in cluster 6 with *Kiss1*-expressing cells distinguished by orange color. The panel on the right plots the number of *Kiss1*-expressing cells in each group with *Kiss1*-expressing cells defined as *Kiss1* LogExp > 0.5 (red dotted line).

The number of *Kiss1*-expressing neurons and the Kisspeptin-like neurons that are not currently expressing *Kiss1* (cells that share a similar gene expression profile with Kisspeptin neurons) were determined by using single-cell RNA sequencing. A total of 4,049 Control and 4,223 SC300 cells were obtained from the scRNAseq analysis of n = 3 pooled hypothalamic tissues (Fig. 6A). After UMAP clustering of single cells, each cluster was identified by marker genes. In the PND 29 hypothalamic tissues of pooled Control and SC300, GABAergic neurons (*Snp25*^+^*Slc32a1*^+^), Glutamatergic neurons (*Snap25*^+^*Slc17a6*^+^), astrocytes (*Agt*^+^), oligodendrocytes (*Pdgfra*^+^, *Fyn*^+^, or *Mobp*^+^), microglia cells (*Tmem119*^+^), ependymal cells (*Tmem212*^+^), non-neuronal cells (*Olig1*^+^), stromal cells (*Mgp*^+^), and macrophages (*Pf4*^+^) were identified (Supplementary Fig. 1).

In the pooled clustering of Control and SC300 scRNAseq data, *Kiss1*-expressing cells were detected in Cluster 6, which also included ependymal cell marker-expressing cells and astrocyte marker-expressing cells (Fig. 6B, Supplementary Fig. 1). Differential expression gene (DEG) analysis was performed to determine the effect of neonatal treatment of EB on the cluster of *Kiss1*-expressing cells. A total of 185 DEGs (adj p < 0.05) were identified in SC300 compared to Control (Fig. 6C). Functional annotation (DAVID Bioinformatics Resources 6.8) of DEGs that were down-regulated in SC300 rats revealed that expression of genes related to aging (10 genes), response to oxidative stress (8 genes), drug metabolism (8 genes), immune responsive (7 genes), and involved in the apoptotic process (7 genes), positive regulation of cell proliferation (7 genes), or positive regulation of neuron projection development (5 genes) were down-regulated in SC300 (Fig. 6D). Interestingly, the projection of gene expression on UMAP clutering revealed that the identified DEGs were down-regulated only in the cluster 6, with no change in the other clusters (Supplementary Fig. 3). Control and SC300 had a total of 307 and 197 cells, respectively, in cluster 6. Among them 68 (22.14%) and 30 (15.23%) cells were *Kiss1*-positive cells in the Control and SC300 rats, respectively (Fig. 6E).

### Neonatal EB-capsule implant had no major impact on body and organ weights, mammary gland formation and blood chemistry

**Figure 7:**
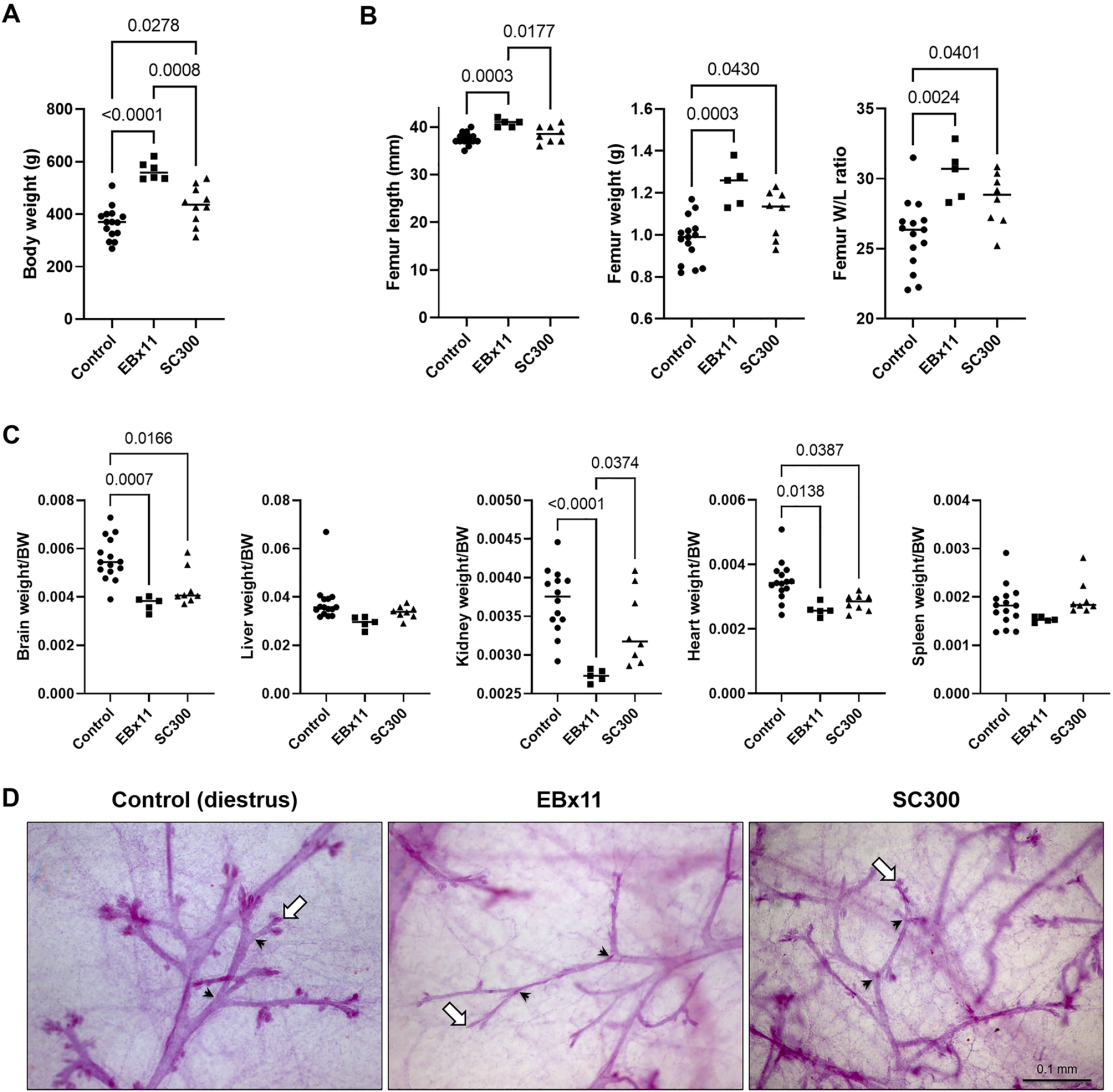
Effects of neonatal estradiol benzoate (EB) on body and organ weights and structure of the mammary gland. Controls received silicone capsules with oil only. The EBx11 group received EB injections on 11 consecutive days from birth (total of 315 µg EB). The EB300 group received a single implant of a silicone capsule containing 300 µg EB. All organs and tissues were collected at PND 178–211 (6–7 months). (**A**) Body weight (BW). (**B**) Femur bone weight, length, and weight/length ratio. (**C**) The weight of essential organs is presented as the ratio of organ weight/BW. (**D**) Mammary gland morphology by whole mount imaging. Arrowheads indicate branching points of mammary ducts. Arrows indicate terminal end buds. Ductal end bud formation appears to be underdeveloped in the EBx11 and SC300 glands, with ductal width also being thinner than in control mammary gland tissues. Error bars = SD. Significant differences between groups are indicated by *P*-values (One-way ANOVA with Tukey post hoc test).

At PND 178–211 (6–7 months), body weights of EBx11 and SC300 rats were 1.55- and 1.19-fold heavier than the controls, respectively (Fig. 7A). SC300 and Controls had similar femur length, but bone weight/length ratios of SC300 and EBx11 were higher than controls (Fig. 7B). Among the major organs, brain, kidney, and heart weight per bodyweight were decreased in EBx11 and SC300 groups, but the liver and spleen weight were not impacted (Fig. 7C). In the liver histology, there were no pathological differences between the four groups (Supplementary Fig. 4). No significant differences in the renal and hepatic serum biochemical biomarkers were noted, except for alkaline phosphatase (ALP), which was increased in both EB treatment groups, and an increase in total cholesterol in the SC300 rats (Supplementary Table 2). The hepatic parenchymal architecture and cellular morphology did not differ between the 3 groups, and the mild increases in serum cholesterol and ALP concentrations could not be correlated with any hepatic histopathology. Mammary glands from the control and SC300 rats were subjected to whole-mount histological analysis and stained with Carmine alum solution to reveal the branching pattern of mammary ducts. In the control rats, mammary tissues revealed normal branching of the ducts and terminal end buds. Mammary duct branching in EBx11 and SC300 appeared to be reduced, and ductal widths and terminal end buds were not as well developed (Fig. 7D). No structural abnormalities, such as dilation of the mammary gland or mammary dysplasia, were observed in the mammary tissues of EB-treated rats. A complete blood count (CBC) was performed to evaluate hemogram and leukogram between treatment and control rats. There were no significant differences in platelet or WBC numbers between groups, whereas the Mean Corpuscular Volume (MCV) in EBx11 was higher than control (Supplementary Table 3).

### Biodegradable EB-microspheres induce sterility in rats with single neonatal injection

**Figure 8:**
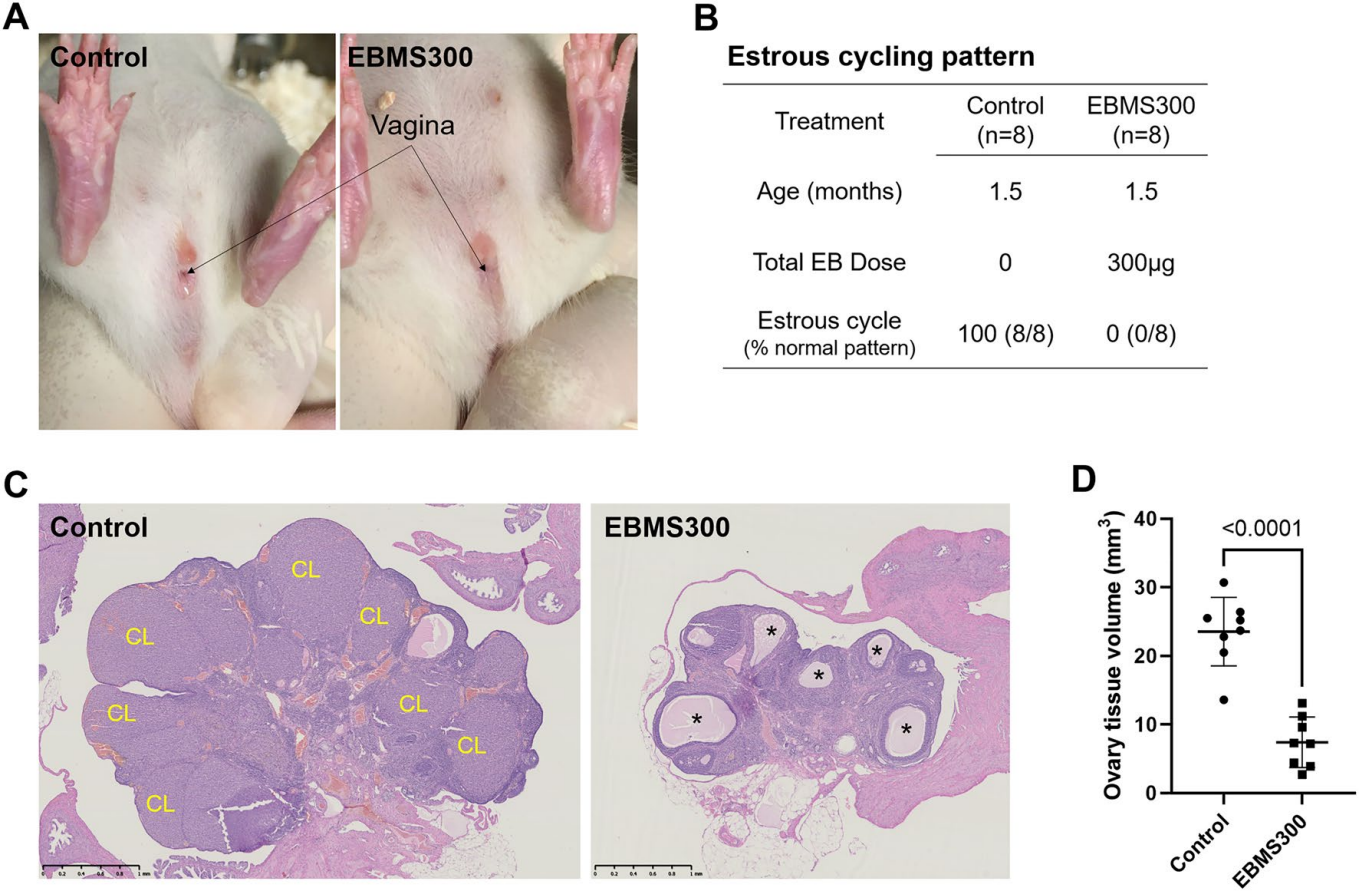
Neonatal injection of biodegradable estradiol benzoate-microspheres (EBMS300) inhibited vaginal opening and ovarian function. Controls were untreated females. The EBMS300 group received a single injection of 300 μg of EB in the reconstituted microspheres. (**A**) Vaginal opening of control and EBMS300 rats until PND 45. (**B**) Estrous cycle of control (n = 8) and EBMS300 (n = 5) rats from PND 44 to 50 (1.5 months). (**C**) Representative histology of ovaries on PND 125. In the control ovary, numerous corpora lutea (CL) are easily recognized as large homogenous structures, which resulted in a greatly enlarged organ. In the EBMS300 ovary, the CL is absent, with only anovulatory follicles (*) being present in a visibly smaller organ. (**D**) Volume of the ovary in control and EBMS300 rats (PND 125). Significant differences are indicated by *P*-values (Student’s *t*-test). Error bars = SD (n = 8).

At 1.5 months of age, control and EBMS300 rats were checked for vaginal opening and evidence of estrous cycling patterns for 2 weeks. EBMS300 females had incomplete vaginal openings and did not show signs of estrus during examination of vaginal cytology, whereas control rats showed normal vaginal openings and displayed regular estrous cycling patterns (Fig. 8). In the first and second rounds of fertility testing, controls had 87.5% fertility, with 12.4 and 10.8 pups/litter, respectively. EBMS300-treated rats did not produce any litters (Table 2). Ovarian histology revealed that EBMS-treated rats had smaller ovaries and were anovulatory, similar to the SC300-treated rats (Fig. 8).

### Neonatal EB-capsule implant inhibits hypothalamic Kisspeptin expression in female dogs

**Figure 9:**
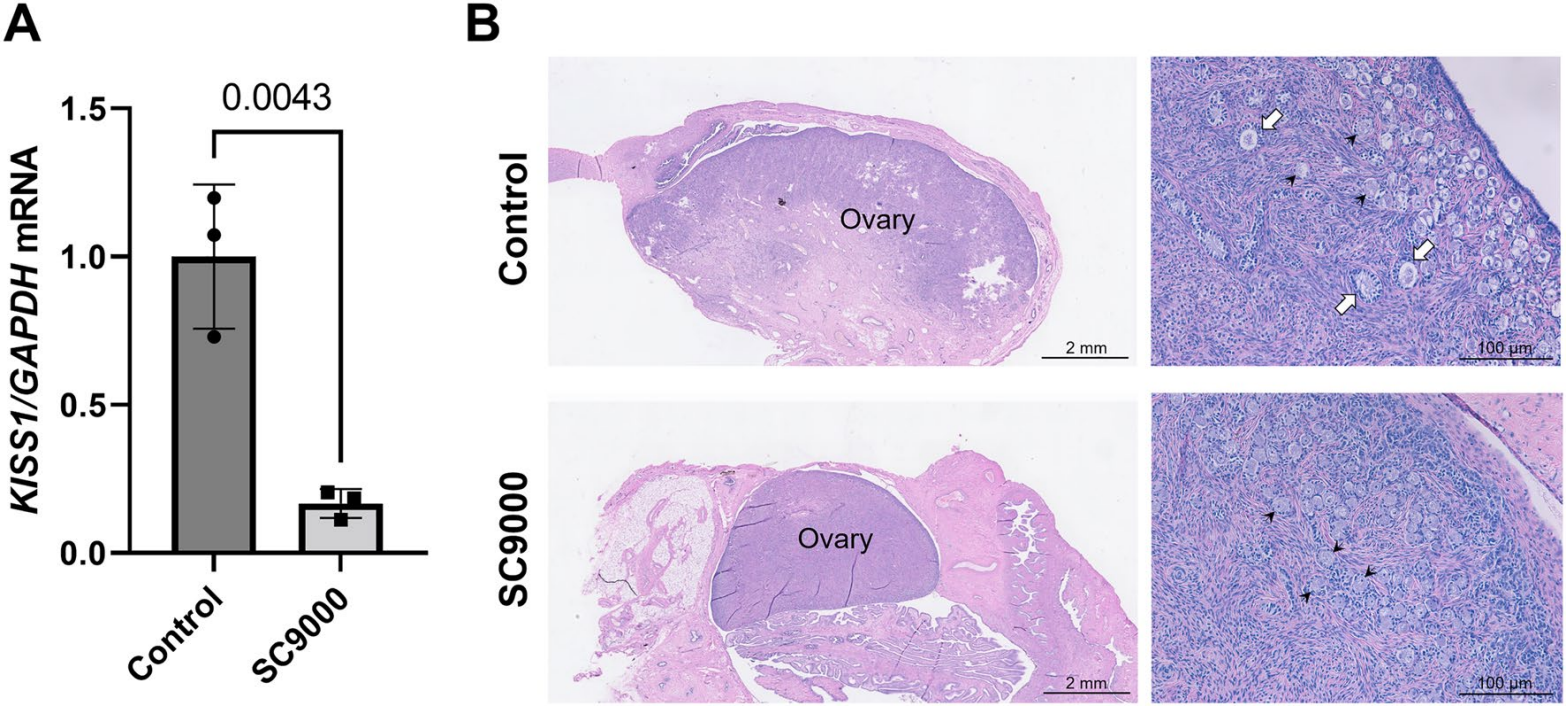
Inhibited hypothalamic *KISS1* expression and ovary development in female Beagle dogs after neonatal implant of estradiol benzoate (EB)-capsule. (**A**) *KISS1* mRNA expression in the hypothalamus of control and SC9000 (9,000 μg EB) dogs on PND 78 (2.5 months). Error bars = SD (n = 3).* P*-values indicate significant differences (Student’s *t*-test). (**B**) Representative histology of control and SC9000 ovaries on PND 78 (2.5 months) (n = 3). Arrowheads and arrows indicate early primary follicles and late primary/secondary follicles, respectively.

The small-scale dog study was performed to provide only a determination of *KISS1* mRNA expression in the hypothalamus, which was the primary target for EB treatment in the rat, and potential downstream effects on the ovary. At PND78, SC9000 dogs (n = 3) had 83% lower *KISS1* mRNA expression compared to that of control dogs (n = 3) (Fig. 9A). At this age, histological observation showed that control dog ovaries had primordial, primary, and late primary/secondary follicles. However, SC9000 ovaries were relatively smaller than those of control’s and no primary and secondary follicles was present, with only primordial follicles being present (Fig. 9B).

## Discussion

In this study, we tested the hypothesis that a permanent infertility (sterility) could be induced in female animals by a transient elevation of estrogen at a neonatal stage using a slow estrogen-release method. Data presented here support the hypothesis, as postnatal treatment-induced elevation of estrogen level using silicone capsule or microspheres induced complete infertility in female rats. Consistent with this finding, neonatal implantation of an EB pellet in the female dog decreased hypothalamic *KISS1* expression, suggesting that the neonatal estrogen treatment may be developed as a non-surgical alternative to surgically sterilizing female dogs.

Previously, delivering EB by daily injections in oil showed that an elevation of circulating E2 levels for at least 2–3 weeks was required for inducing sterility in female rats^27,46^. However, such treatment method would be impractical for use in the veterinary profession and daily injections would maintain a rather high concentration of E2 over the entire treatment period, which could lead to adverse health effects. Therefore, we designed EB delivery devices (silicone capsules and microspheres) to release EB over a 2–3-week period by a single treatment, with slight differences in their pharmacokinetics. When implanted neonatally, the SC300 rats maintained serum E2 level of 200 pg/mL or higher for over 2 weeks, and in the hypothalamus E2 levels were 10 times higher than in the blood. However, the hypothalamic elevation of E2 was short-lived and decreased rapidly to the control level within a week while the blood level of E2 was still maintained at > 200 pg/mL. Hypothalamus is known to contain a higher E2 concentration than blood^47,48^ and to accumulate E2 in neonatal stage in particular^49^. Therefore, the unexpectedly high E2 level observed in the hypothalamus of SC300 implanted animals is thought to be achieved by an innate E2 accumulating physiology in the hypothalamus. The rapid decline of hypothalamic E2 is likely due to the ability of the hypothalamus to actively remove the steroid. Generally, in the adult female, local E2 levels are an outcome of gonadal E2 synthesis and its metabolic degradation or its conversion to less-active forms of estrogen, such as estrone (E1) and estriol (E3)^50^. The oxidative 17β-hydroxysteroid dehydrogenases (17β-HSDs) such as 17β-HSD4 and 17β-HSD8 are known to play a key role in this metabolic process^50–52^. Our hypothalamic scRNAseq data showed an abundance of cells that express oxidative 17β-HSDs-positive, but only scant reductive 17β-HSDs-expressing cells (Supplementary Fig. 5). Thus, E2 removal was likely active in hypothalamic cells at this neonatal age. Based on these findings, future investigation of their potential enzymatic activity and role in the clearance of E2 from the hypothalamus is warranted. In the meantime, the mRNA expression levels of 17β-HSDs in the hypothalamus of the PND 29 were not different between Control and SC300, indicating there was likely no lasting impact on the expression of those genes by the neonatal hypothalamic E2 elevation.

It has previously been reported that direct exposure of the hypothalamus via E2 micro pellets or infusion in the neonate was sufficient to sterilize female rats, while implanting the same pellets or injecting the equivalent dose of E2 subcutaneously was not effective in inducing sterility^53,54^. However, in our current study, administering a single dosing of EB subcutaneously was able to induce sterility, most likely due to the ability to induce a significant elevation of E2 in the hypothalamus for a longer period of time using the extended-release methods. Female rats that were neonatally treated with EB implant or microspheres did not exhibit any sign of pubertal onset nor display estrous cyclicity. Of note, treatment of those rats with a KISS analog instantly elevated circulating LH to the level that was comparative to intact animals, indicating that GnRH neurons and pituitary gonadotrophs retain their functional responsiveness.

The onset of puberty is triggered by the functional crosstalk among the organs of hypothalamic-pituitary-ovarian axis^55–57^, especially through the activation of GPR54 signaling by KISS1 secreted from Kisspeptin neurons of the hypothalamus^58–62^. In the hypothalamus of primates and rodents, *Kiss1* mRNA expression increases toward the onset of puberty^63–65^. Indeed, *Gpr54* knockout (KO) mice do not undergo puberty nor show estrous cyclicity but show under-developed ovaries, sub-physiological level of serum sex steroid and do not ovulate^60,66,67^. These phenotypic characteristics of *Gpr54*KO mice are nearly identical to those of the SC300-treated female rats, suggesting a disrupted KISS1-GPR54 system. However, the SC300 rats had a partially opened vagina, unlike the *Kiss1*KO mice in which the vagina was completely closed at puberty^21,68^. This difference was likely due to neonatal SC300-treatment having only a partial effect on the Kisspeptin neurons, while in the *Kiss1*KO mouse, there was complete absence of KISS1-driven GPR54 activation. Hence, the partially formed vaginal opening could be a consequence of reduced, yet not entirely diminished, hypothalamic Kisspeptin levels resulting from EB treatment. This was further supported by the fact that ovaries in the *Kiss1*KO female mice displayed follicles up to the antral stage of development, but no preovulatory follicles were observed^69^, whereas in the present study ovaries from all neonatal EB treatments had all stages of follicles, including antral follicles, but there was no formation of a corpus luteum, indicating a total absence of ovulation. Thus, the neonatal EB treatments as tested inhibited ovulation, but did not impair folliculogenesis. It is reasonable to hypothesize that while EB produced a reduction in KISS1 expression, it did not completely eliminate its expression and activity, thus allowing for antral follicle development, but disrupting possibly the required surge in LH, as previously observed^27,70^. The impact of SC300 treatment on neonates was distinct in terms of its effect on the ovaries; however, its influence on uterine weight was limited. Notably, smaller uteri were observed only in EBx11 rats compared to the control group, which seems to be a result confined to cases where a high dose of EB was administered over a short period. Similarly, a decrease in uterine weight was confirmed in rats that received 10 µg of E2 daily for 5 days post-birth^71^. These findings suggest that SC300 has a limited direct effect on the uterus compared to EBx11, which is thought to be due to the slower and declining drug release compared to EBx11.

ARC Kisspeptin neurons primarily serve the role of GnRH pulse generator, while AVPV Kisspeptin neurons induce the mid-cycle LH surge and subsequent ovulation^19^. In the adult, these separate populations show differential regulation by E2^72^. In the neonate, little is known of their regulation; however, ovaries in the SC300-treated rats did not form a CL, indicating that ovulation did not occur. Thus, in addition to decreasing the ARC kisspeptin neurons, the study also supports a previously reported ability of neonatal EB treatment to inhibit the AVPV Kisspeptin neurons. The previous study found that a single dose of EB during the neonatal period could suppress KISS1 expression in the AVPV, while an inhibition of KISS1 expression in the ARC required multiple EB treatments between PND 0–10^27^. As a result, it has been suggested that AVPV Kisspeptin neurons have a higher sensitivity to EB treatment than ARC Kisspeptin neurons. Thus, it is posited that the decreased KISS1-ir in the ARC, which has relatively low sensitivity to neonatal E2 treatment, would be accompanied by a decrease in Kisspeptin expression in the AVPV, ultimately leading to infertility. Therefore, KISS1 expression was assessed in the ARC by the intensity of KISS1-ir. The results revealed that the EB treated rats had lower KISS1-ir in the ARC on PND 29. However, it was important to determine if this was simply an inhibition of neuronal activity or if the reduction represented a reduced number of Kisspeptin neurons. The reduction in activity could be interpreted as a temporary effect, but the loss of neurons would likely result in a permanent effect.

The transcriptome analysis of hypothalami found that EB-treatment decreased the mRNA expression of multiple genes known to be involved in regulating neuronal development, neuron projection, and cell proliferation, specifically in the cluster that includes *Kiss1*^+^ cells (Fig. 6C, Supplementary Fig. 3). Minabe et al. (2017) suggested that estrogen receptor α and/or β (ESR1 and ESR2) likely mediate the inhibitory action of E2 on Kisspeptin neuron differentiation and viability^27^. This finding raises the possibility that estrogen may directly inhibit the development or survival of the Kisspeptin-lineage cells via interaction with ESRs. This hypothesis is supported by a previous finding that repeated neonatal EB treatments decreased the number of not only *Kiss1*-expressing cells, but also *Pdyn*- or *Tac3*-expressing cells in the hypothalamic ARC region^27^. Previous studies have suggested that the number of Kisspeptin neurons decreases via apoptosis or inflammatory responses, as a result of an increase in E2 levels, specifically in males during the neonatal period. Males have higher BAX, proapoptotic Bcl-2 (B-cell lymphoma 2)-associated protein, expression levels than females in the developing AVPV region^73,74^, and this is thought to be a reason for the sexual difference in Kisspeptin populations between males and females. Indeed, the *Bax*KO eliminated the sex difference in total number of AVPV cells^75^. More importantly, *Bax*KO increased the number of AVPV and ARC Kisspeptin neurons in both sexes^76^. E2 increases neuronal inflammation by mediating the induction of COX-2 and subsequently, PGE2 can also affect neuronal apoptosis as seen in AVPV kisspeptin neurons during exogenous E2 administration^77^. Although the mechanism that regulates apoptosis in Kisspeptin neurons by estrogen is not clearly known, the neonatal E2 treatment-induced sterility suggests that Kisspeptin-lineage neurons were removed by E2/ESRs-mediated apoptosis. Additionally, our study has shown a decrease in gene expression related to the development of neurons in rats treated with SC300. This suggests that developmental inhibition of Kisspeptin neuron by E2 could also be proposed as a new mechanism in E2-induced sexual dimorphism in the hypothalamus. Based on this working principle that EB could suppress hypothalamic Kisspeptin neuron development or survival, the hypothetical window for induction of sterility by EB would be limited to neonatal ages, and possibly extending to an early prepubertal period. Although it is clear that EB treatment in neonates decreased the number of hypothalamic Kisspeptin neurons, this treatment would not be successful if given at a pubertal or post-pubertal age, as the Kisspeptin neuron population would already be established. Adult treatment would likely reduce LH levels^78^, but this would only be temporary and not induce sterility. However, further research involving EB treatment in post-pubertal rats is needed to confirm this.

Induced infertility in the present study shares a working principle with a previous report that neonatal female rats treated with EB show continuous anestrus due to suppression of ARC *Kiss1* expression and the LH pulse^27^. However, it remained unclear whether these results were due to abnormalities in the function of Kisspeptin neurons or GnRH neurons. Therefore, to verify whether there was a decrease in GnRH neuron function due to EB in treated rats, we performed a KP-10 challenge experiment. If GnRH neurons were still responsive to ligand stimulation and capable of downstream activity, KP-10 should cause the release of LH. KISS1 acts on downstream GnRH neurons resulting in the release of GnRH, which in turn stimulates the synthesis and release of LH from pituitary gonadotrophs^79^. SC300 treated females that were given KP-10 (a kisspeptin analog) showed a robust increase in serum LH, similar to controls, demonstrating that the neonatal estrogen treatment had not produced a permanent effect on the GnRH neurons or pituitary gonadotrophs, but rather was focused on the kisspeptin neurons in the hypothalamus. Thus, during estrus the control pituitary would be able to respond to the normal secretion of KISS1, while the SC300 pituitary, although capable of responding, would not do so because of the KISS1 deficiency. However, the underlying mechanisms by which neonatal treatment with exogenous E2 decreased the number of hypothalamic Kisspeptin neurons remains unknown.

Exposures to exogenous estrogens during the neonatal period have for many years been suspected of producing adverse effects on skeletal development and inducing dilation of the mammary gland ductules, and endbuds^80,81^. With such concern in mind, the effects of EB treatment of neonatal rats were assessed, but no abnormalities were found in the femur bones and the mammary gland had a reduction in branching and ductal widths, with underdevelopment of the endbuds. Furthermore, the present study found no health-related problems or major gross or histopathological changes in major organs outside of the reproductive system. The lack of adverse effects was likely due to the slow release of EB from the implant and declining concentration of E2 over time, limiting the adverse impact of EB in other growing organs. In the current study, there were no differences between control and EB-treated groups in liver weights, gross morphology, and histopathology. The increased serum ALP concentration in the EBx11 and SC300 rats might be due to an upregulation of the ALP bone-isoenzyme because of accelerated bone growth. It is also postulated that the mild hypercholesterolemia in the SC300 rats was due to dyslipidemia induced by an E2-deficient state as previously documented in rodents and postmenopausal women^82^.

However, it is known that loss of reproductive function alone may result in some changes in blood chemistry. For example, it is well documented that routine surgical sterilization by OVX can result in increased levels of ALP, ALT, AST, and cholesterol, compared to sham-operated animals^83,84^. Therefore, we compared serum chemistry changes induced by EB treatment with those seen in OVX rats. By comparison, SC300-treated rats only had a mild increase in serum ALP concentration (Supplementary Table 2). More importantly, SC300 treatment did not result in hepatitis and hepatic steatosis, whereas OVX can contribute to these changes^83,85^. The increase in body weight after EB treatment compared to control rats mimics the weight gain that occurs in OVX rats^86^. This indicates that loss of gonadal function and steroidogenic capacity can alter metabolic activity and fat accumulation as the animal reaches puberty. Indeed, OVX rats showed increased body weight, lower liver weight relative to body weight, lower kidney weight relative to body weight, and lower spleen weight relative to body weight compared to the control group and SC300 rats (Supplementary Fig. 6). Although ALP and serum cholesterol levels increased to similar extents in both SC300 and OVX groups compared to the control group, these results indicate that, at least in the examined parameters, SC300-treated rats were healthier than OVX rats in terms of obesity risk.

The sterilization method using neonatal EB delivery has benefits over surgical sterilization. The ovaries continue to produce E2 for several months, allowing the animals to benefit from circulating E2 that is requisite for musculoskeletal growth. The presence of the ovary and its ability to produce E2 in the SC300 rats produced a better physiological outcome than OVX. It is noteworthy also that SC300 rats showed a smaller increase in body weight than the EBx11 rats, indicating that slow release of EB, with declining serum levels over a short period of time, could help prevent fat accumulation, in contrast to the OVX animals. Sustained release of EB is safe and has advantages over OVX for sterilization. Estrogen and its derivatives are commonly used in human and animal medicine, especially in women, where estradiol, estradiol valerate, estradiol acetate, estradiol cypionate and other synthetic estradiol compounds are used for preventing or reducing menopausal symptoms^87–89^. Estrogen is used in dogs for the treatment of urinary incontinence and hormone replacement therapy^89–92^. In food production animals such as beef cattle, EB implants are approved for improving feed efficiency^93^. In this study, no major health concerns were found in female rats receiving neonatal EB treatment, but extensive work is needed to address potential safety concerns in other species.

Steroids can be administered in mammals, including humans, through various delivery systems, such as pills, oil carrier injections, tampons, transdermal patches, pellets of various compositions, infused microspheres, and implants of cured silicone elastomers^94–97^. One method involves mixing steroids with silicone rubber, with and without the use of membranes^98^. Another method uses Silastic tubing to implant concentrated steroid crystals or steroid mixed with liquid silicone elastomer in discrete lengths for control of dosage^31,96^. PLGA-based microspheres systems provide an advanced drug delivery technology approved by the US Food and Drug Administration as a biodegradable polymer for hormone therapy in humans; e.g. Zoladex^99^. The active ingredients, which are physically entrapped in the PLGA-drug complex, are released through esterase-mediated hydrolytic degradation of the PLGA structure and excreted from the body, which results in a complete removal of the polymer^100–102^. Both the silicone capsule and PLGA microsphere systems are efficient for drug delivery, but there may be hesitancy to use silicone capsules in pets or food production animals because silicone is non-degradable and would remain permanently after the drug is released. Manufactured silicone implants would also less flexible for adjusting the dose according to body weight. For those reasons, EB-containing biodegradable PLGA microspheres were designed and tested and found to be as effective as silicone capsules. A prior in vivo study observed that PLGA microspheres containing steroids smaller than 10 µm in size continued to release the drugs for 21 days^103^. This would imply that using PLGA microspheres to deliver EB could increase hypothalamic E2 levels in a pattern similar to that found with silicone capsules and induce infertility in female rats, similar to SC300. Indeed, the data demonstrated that PLGA microspheres could serve as a biodegradable material for EB delivery.

Because kisspeptin neurons of ARC have been reported in other mammals, including sheep, pigs, dogs, cats, goats, cattle, guinea pigs, and monkeys, it is possible this sterilization method, with some modification of the EB release pattern, could have universal application for several species including zoo and pet animals^104^. Kisspeptin neurons have been reported in the female dog^105^; therefore, we performed a pilot study in neonatal female Beagle pups. Similar to the rat, neonatal treatment with EB (equivalent to 22.5 mg/kg BW) in the female dog decreased *KISS1* expression in the hypothalamus and inhibited or delayed ovarian development at 3.5 months of age, as evidenced by the absence of primary/secondary follicles. This indicates that neonatal treatment of dogs is worth investigating as an alternative to surgical sterilization (spay or neuter). In future studies, we will expand this study to validate the efficacy and safety of neonatal EB treatment through long-term surveillance during the entire dog's lifespan. This technology may not be applicable to animals of all ages, but could be used as a sterilization technique for animals born in shelters. Based on the mechanism of sterility induction through the suppression of Kisspeptin neuron development and the decrease in cellular *Kiss1* expression, it is likely that infertility in the male could also be induced by neonatal exposure to EB. Indeed, similar to female rats, neonatal EB treatment in the male suppressed ARC *Kiss1*, thereby disrupting the onset of puberty, which resulted in testicular atrophy due to reduced levels of testosterone^106^.

## Summary and conclusions

A single neonatal subcutaneous administration of an estrogen via slow-release methods induced permanent infertility (sterility) in female rats. This supports the idea that a supraphysiological, but temporary, increase in E2 levels during postnatal development can disrupt the HPG axis by permanently inhibiting Kiss1 expression, while leaving GnRH neurons and gonadotrophs intact. Neonatal treatment of female rats with EB reduced the number of Kisspeptin neurons and their immunoreactivity in the hypothalamus, which resulted in permanent non-ovulation. Repeated dosing of the animals was not necessary, as a single capsule implant of EB or injection of EB-microspheres was sufficient to induce anovulation. Health safety parameters were found to be similar to surgical ovariectomy. Preliminary testing in female dogs showed similar suppression of KISS1 expression in the hypothalamus. Therefore, further development of this estrogen-treatment method for sterilization in domesticated animals, such as female dogs and cats is worthy of investigation. Such research should begin by determining the appropriate age range for EB treatment to induce sterilization in dogs and cats.

## Supplementary Information

Supplementary Information.

## Data Availability

The code and test sets are available in the github repository https://github.com/cjpark85/Rat_Hypothalamus_scRNAseq and the NCBI GEO database (GSE221002).
