# Supplementary material for: Single neonatal estrogen implant sterilizes female animals by decreasing hypothalamic KISS1 expression
Source: Sci Rep. 2023 Jun 14;13:9627. doi: 10.1038/s41598-023-36727-8 (PMC10267159; doi:10.1038/s41598-023-36727-8)
Supplement: Supplementary file 1 — Supplementary Information. [file 41598_2023_36727_MOESM1_ESM.pdf]

Supplementary Figures and Tables

Single neonatal estrogen implant sterilizes female animals by decreasing hypothalamic KISS1 expression

Chan Jin Park[1,2], Shiori Minabe[3], Rex A. Hess[2], Po-Ching Patrick Lin[1], Sherry Zhou[2], Shah Tauseef Bashir[1], Radwa Barakat[1,4], Arnon Gal[5], and CheMyong Ko[1,2]

[1]Department of Comparative Biosciences, College of Veterinary Medicine, University of Illinois at Urbana-Champaign, Urbana, IL 61802, USA.

[2]Epivara, Inc., Champaign, IL, 61820, USA.

[3]Iwate Tohoku Medical Megabank Organization, Iwate Medical University, Iwate, 028-3694, Japan.

[4]Department of Toxicology and Forensic Medicine, Faculty of Veterinary Medicine, Benha University, Qalyubia 13518, Egypt.

[5]Department of Veterinary Clinical Medicine, College of Veterinary Medicine, University of Illinois at Urbana-Champaign, Urbana, IL 61802, USA.

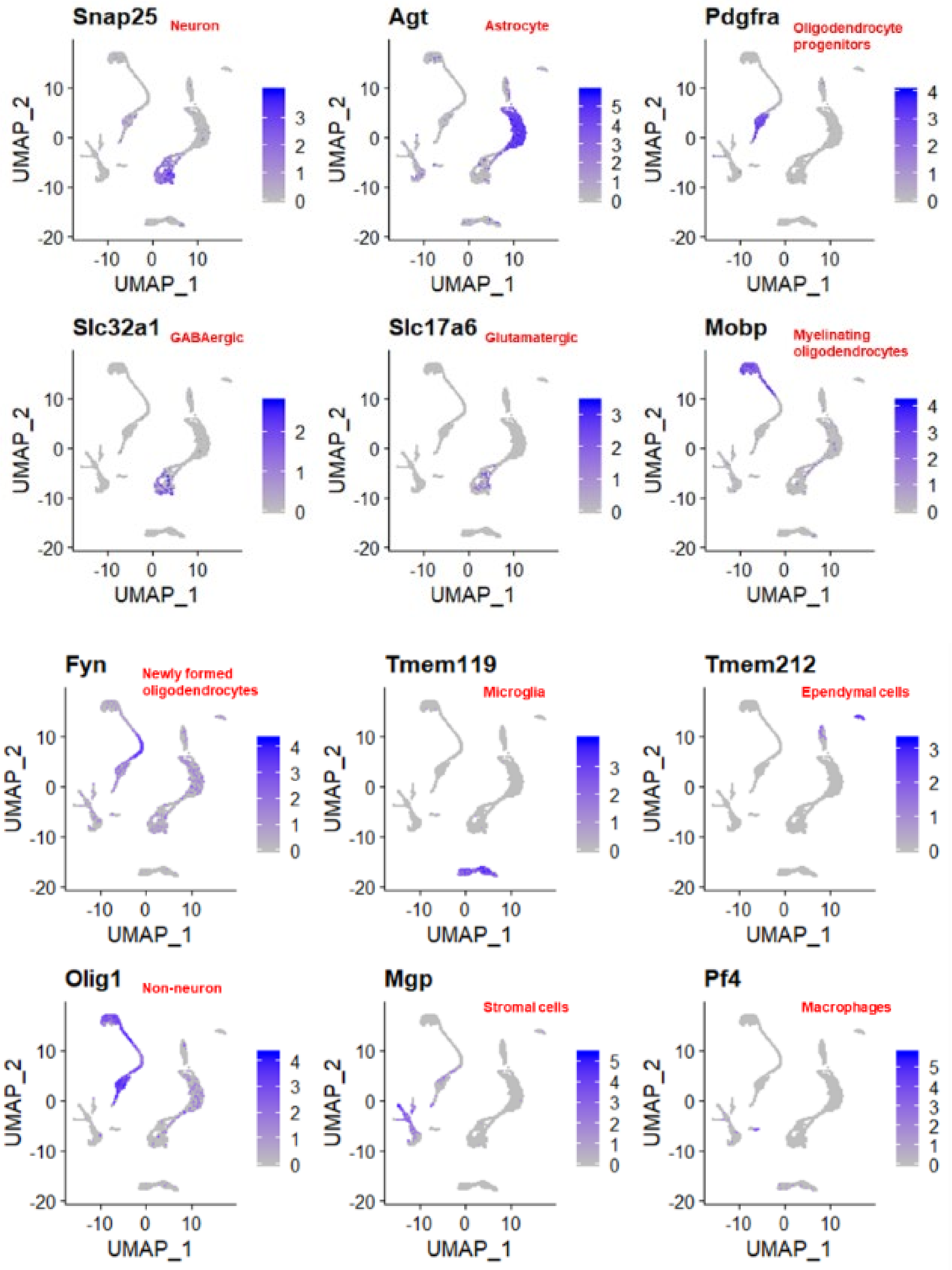
Snap25
Neuron
Agt
Astrocyte
Pdgfra
Oligodendrocyte progenitors
Slc32a1
GABAergic
Slc17a6
Glutamatergic
Mobp
Myelinating oligodendrocytes
Fyn
Newly formed oligodendrocytes
Tmem119
Microglia
Tmem212
Ependymal cells
Olig1
Non-neuron
Mgp
Stromal cells
Pf4
Macrophages
UMAP_1
UMAP_2

Supplementary Figure 1. Cluster identification in hypothalamic cells by single-cell RNA sequencing. Hypothalamic cells of n=3 Control or SC300 rats were pooled and UMAP clustered together. The following markers were localized in the UMAP plots of hypothalamic cells from Control and SC300 rats: Neuron marker (*Snap25*), Astrocyte marker (*Agt*), Oligodendrocyte markers (*Pdgfra, Fyn, Mobp*), Microglia marker (*Tmem119*), Ependymal cell marker (*Tmem212*), GABAergic neuron marker (*Slc32a1*), Glutamatergic neuron marker (*Slc17a6*), Non-neuronal cell marker (*Olig1*), Stromal cell marker (*Mgp*), Macrophage marker (*Pf4*). Relative expression level is shown by color density, with the darkest blue corresponding to highest expression.

Age | **Estrous cycling pattern**

2.5 M

Treatment	Control (n=8)	SC300 (n=5)
Age (months)	2	2
Total EB Dose	0	300ug
Estrous cycle (% normal pattern)	100 (8/8)	0 (0/5)

Control **SC300**

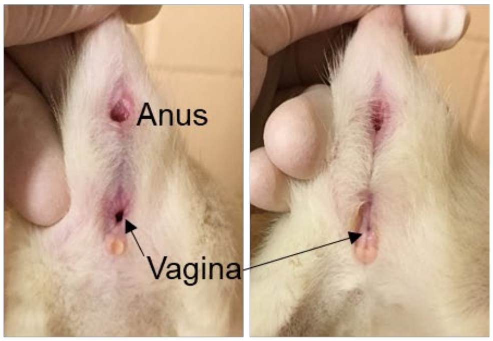

* Vagina is closed in the adulthood.

11 M

Control **SC300**

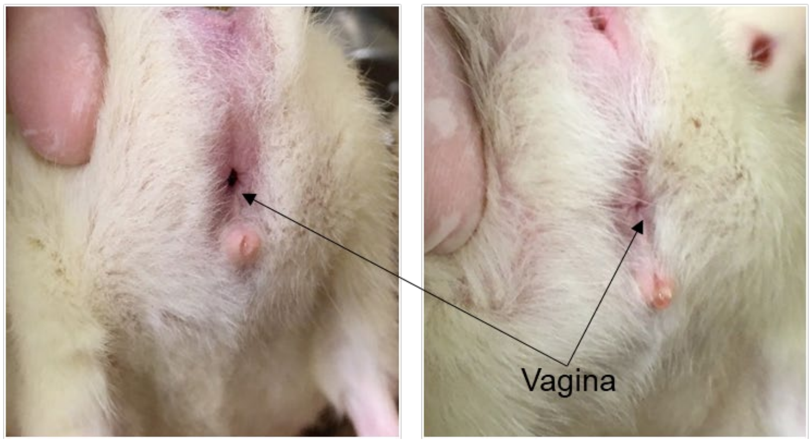

Supplementary Figure 2. Estrous cycle and vaginal opening of control and SC300 female rats at PND 46 (2.5-month-old) or PND 335 (11-month-old) after birth.

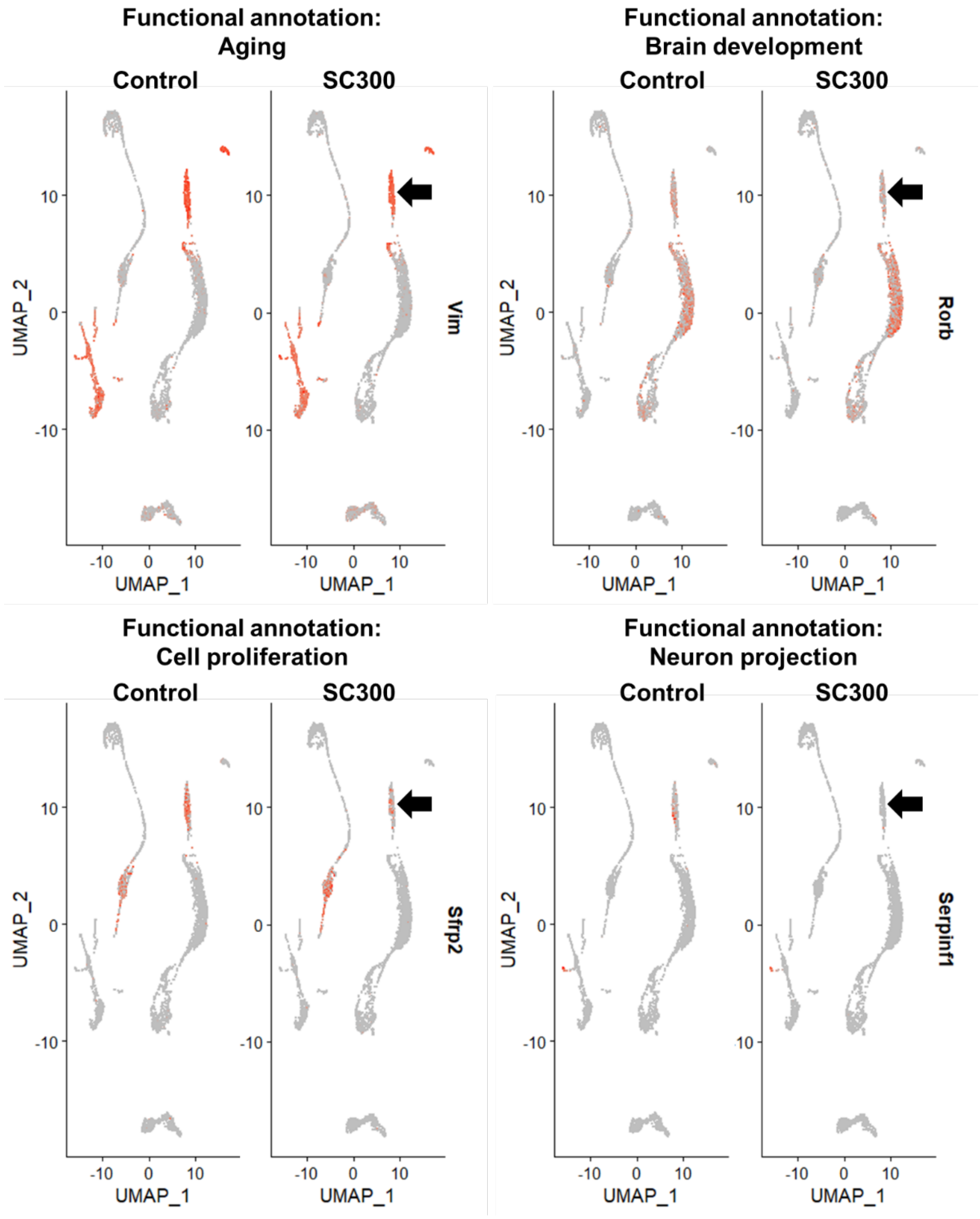

Supplementary Figure 3. Visualization of gene expression in UMAP clustering of cells from the hypothalamus in Control and SC300 female rats. Expression patterns for representative down-regulated DEGs in SC300 rats include the following: *Vim*, *Rorb*, *Sfrp2*, and *Serpinf1.* Listed genes are down-regulated specifically in the clusters that have *Kiss1*$^{+}$ cells (arrows).

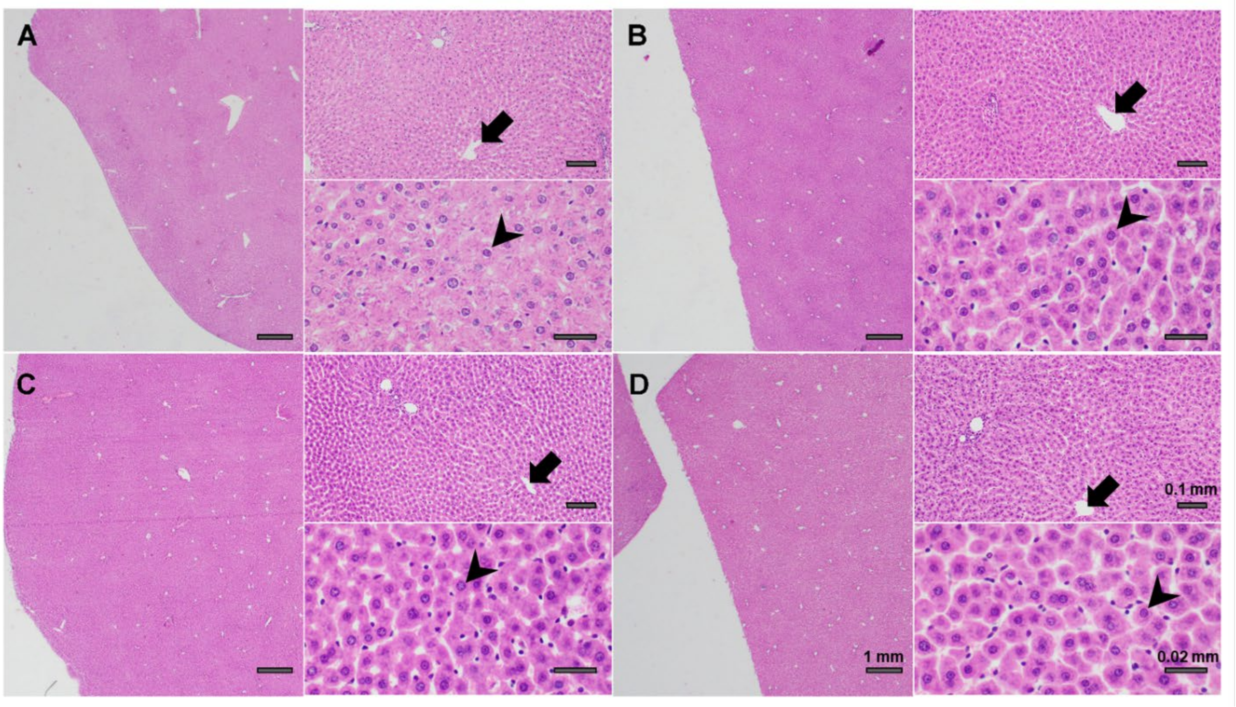

Supplementary Figure 4. Representative liver histology in Control (A), EBx11 (B), SC30 (C), and SC300 (D) groups. Arrows, central vein, Arrow heads, single hepatocyte.

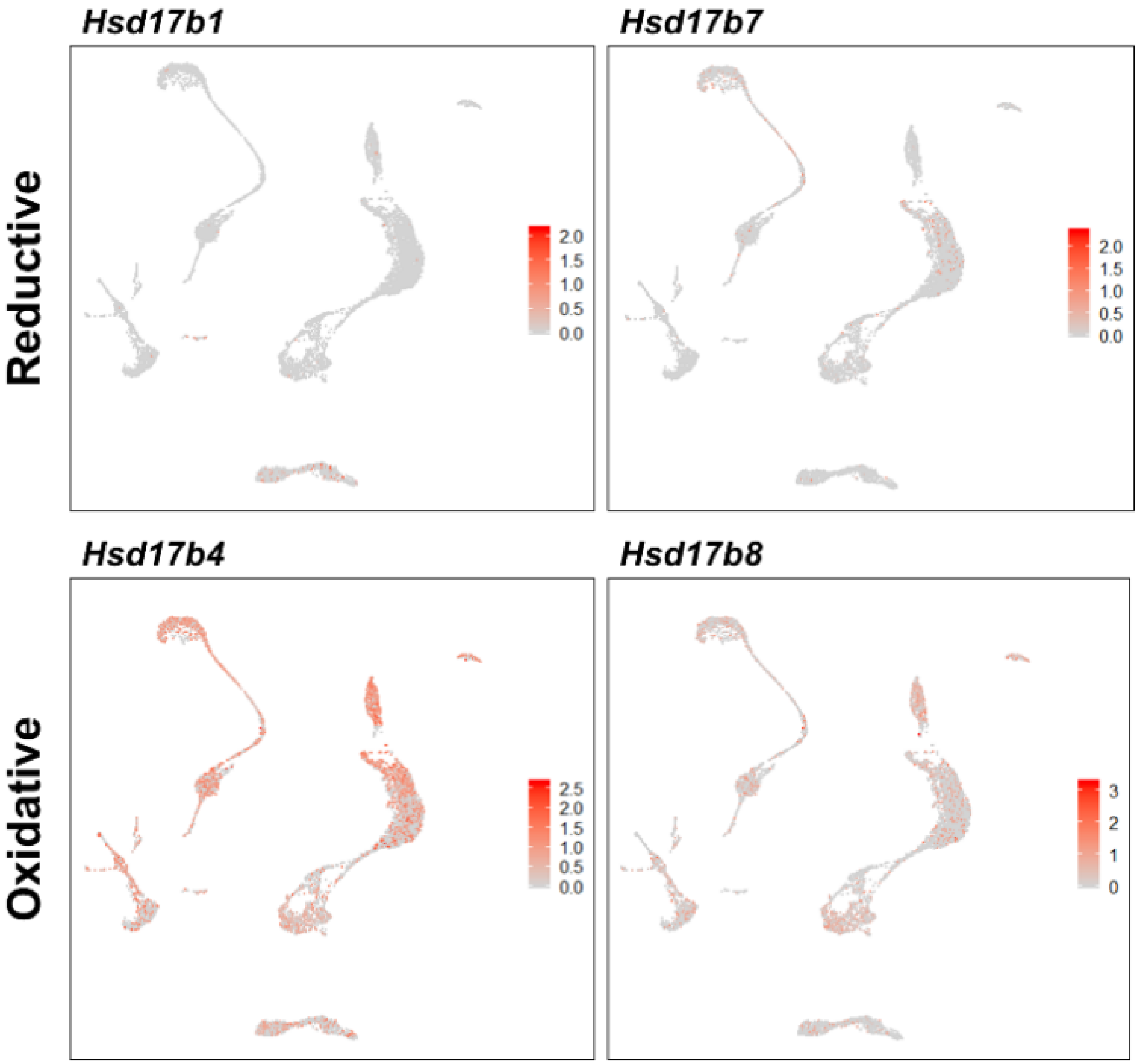

Supplementary Figure 5. Visualization of reductive and oxidative 17β-HSDs expressions using UMAP clustering of hypothalamic cells. The relative expression level of each gene was presented by the color gradient (gray-red).

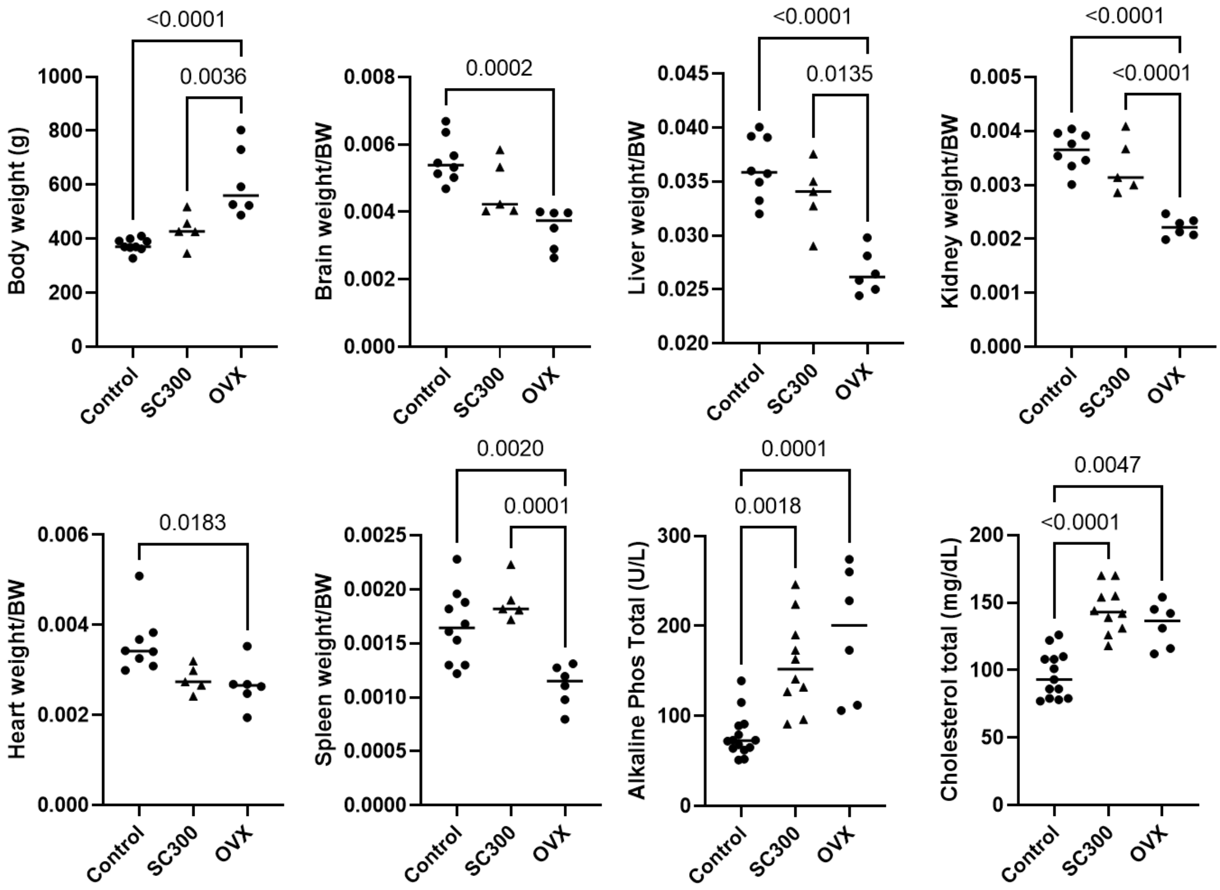

Supplementary Figure 6. Body weight (BW), brain weight/BW, liver weight/BW, kidney weight/BW, heart weight/BW, spleen weight/BW of control (n=8), SC300 (n=5), and ovariectomized (OVX; n=6) rats, respectively, and Alkaline Phos Total (ALP) and cholesterol levels of control (n=14), SC300 (n=10), and ovariectomized (OVX; n=6). All animals were euthanized at 6 months of age. Error bars = SD. Significant differences between groups are indicated by *P*-values (One-way ANOVA with Tukey post hoc test).

Supplementary Table 1. Serum and hypothalamic E2 levels in female rats (mean ±SD).

Day	Serum E2 levels (pg/mL)				Hypothalamic E2 levels (μg/mL)			
	Control	n	SC300	n	Control	n	SC300	n
**0**	4.0 ± 3.7	4			1.29 ± 1.0	4		
**1**	6.1 ± 2.3	4	712.3 ± 81.3	6	1.64 ± 0.3	4	44.1 ± 9.6	4
**2**	14.7 ± 12.6	6	737.9 ± 54.2	7	1.06 ± 1.1	5	41.2 ± 9.6	7
**3**	16.8 ± 10.2	6	512.2 ± 103.7	8	1.38 ± 1.0	6	31.3 ± 14.6	3
**4**	3.4 ± 1.6	4	634.6 ± 90.1	4	1.54 ± 0.2	4	4.4 ± 0.9	4
**7**	3.3 ± 1.4	4	496.7 ± 86.6	4	1.37 ± 1.8	4	1.8 ± 0.4	3
**10**	7.3 ± 2.9	3	462.4 ± 27.7	4	1.28 ± 1.3	3	2.5 ± 2.3	3
**21**	4.0 ± 4.1	6	89.5 ± 6.8	4				
**30**	2.8 ± 2.7	6	33.6 ± 8.5	4				

Supplementary Table 2. Serum biochemical parameters in 6-7-month-old female rats (mean ±SD).

Criteria	Control (n=14)	EBx11 (n=6)	SC300 (n=10)	OVX (n=6)
Creatinine (mg/dL)	0.5 ± 0.06	0.5 ± 0.08	0.5 ± 0.12	0.4 ± 0.05
BUN (Urea) (mg/dL)	20.1 ± 5.53	23.3 ± 5.26	23.5 ± 2.66	18.2 ± 0.98
Total Protein (g/dL)	7.7 ± 0.43	7.1 ± 0.15	7.1 ± 0.48	6.7 ± 0.27[a]
Albumin (g/dL)	4.1 ± 0.31	3.6 ± 0.09	3.6 ± 0.26	3.7 ± 0.10
Globulin (g/dL)	3.5 ± 0.27	3.5 ± 0.18	3.5 ± 0.46	2.9 ± 0.21
Albumin/Globulin Ratio	1.2 ± 0.11	1.1 ± 0.08	1.1 ± 0.14	1.3 ± 0.10
Calcium (mg/dL)	12.5 ± 0.67	12.4 ± 0.27	12.8 ± 0.98	12.9 ± 0.36
Phosphorus (mg/dL)	9.0 ± 0.72	9.0 ± 0.73	9.5 ± 2.99	8.5 ± 0.79
Sodium (mmol/L)	145.1 ± 2.20	146.8 ± 1.92	148.4 ± 3.85	147.5 ± 0.55
Potassium (mmol/L)	7.3 ± 1.24	7.8 ± 0.51	7.3 ± 1.73	6.2 ± 0.57
Chloride (mmol/L)	97.5 ± 2.18	100.0 ± 1.10	100.3 ± 2.00	99.5 ± 1.05
Glucose (mg/dL)	286.4 ± 90.21	320.3 ± 58.81	306.1 ± 42.39	367.2 ± 46.23
ALP (U/L)	78.1 ± 24.17	149.2 ± 58.34[a]	143.4 ± 43.35[a]	192.2 ± 73.21[a]
ALT (SGPT) (U/L)	52.3 ± 14.72	62.5 ± 16.07	71.2 ± 43.85	54.8 ± 7.28
AST (SGOT) (U/L)	98.6 ± 41.85	113.8 ± 31.89	132.3 ± 85.3	86.8 ± 26.13
GGT (U/L)	0.7 ± 0.63	1.5 ± 0.84	1.4 ± 1.49	0.0 ± 0.00
Total Bilirubin (mg/dL)	0.2 ± 0.05	0.2 ± 0.05	0.2 ± 0.00	0.2 ± 0.05
Cholesterol total (mg/dL)	104.4 ± 34.34	134.2 ± 17.82	140.6 ± 16.49[a]	133.3 ± 16.73[a]
Triglycerides (mg/dL)	202.0 ± 91.46	195.3 ± 83.76	159.8 ± 32.25	356.0 ± 72.55[a]
Bicarbonate (mmol/L)	23.9 ± 3.70	24.7 ± 4.82	24.8 ± 6.42	30.2 ± 3.66
Anion Gap	31.0 ± 4.30	30.0 ± 3.54	30.4 ± 10.27	24.2 ± 4.07

[a] Significantly different from Control (One-way ANOVA, P<0.05)

Supplementary Table 3. Complete blood count parameters for 6-7-month-old female rats (mean ±SD).

Criteria	Control (n=12)		EBx11 (n=5)		SC300 (n=7)		OVX (n=6)	
Red Blood Cells (x10^6/μl)	7.63	±0.47	8.01	±0.44	7.83	±0.70	8.30	±0.51
Hemoglobin (g/dL)	13.98	±0.99	15.12	±0.61	14.81	±1.39	15.48	±0.69
Hematocrit (%)	43.16	±2.81	47.36	±1.37	46.04	±4.27	47.80	±0.02
Platelets (x10^3/μl)	854.08	±91.20	759.4	±69.76	932.57	±92.29	976.6	±54.78
MCV (fl)	56.55	±1.53	59.21	±2.34[a]	58.80	±1.68	57.56	±1.18
MCHC (g/dL)	32.34	±0.50	31.92	±0.62	32.20	±0.50	32.42	±0.55
White Blood Cell Count (x10^3/μl)	7.99	±2.80	8.33	±2.38	10.65	±2.56	8.06	±3.39

[a] Significantly different from Control (One-way ANOVA, $P<0.05$)
